# Multifunctional Hydrogels: Rational Design and Application in Regenerative Medicine

**DOI:** 10.1002/mco2.71046

**Published:** 2026-10-06

**Authors:** Linyang Song, Epiphane K. Silli, Yazhen Xu, Huixia Lv, Ruoxian Song

**Affiliations:** ^1^ Department of Orthopedics The 960th Hospital of PLA Jinan China; ^2^ Qilu Hospital of Shandong University Jinan China; ^3^ Department of Pharmaceutics School of Pharmacy China Pharmaceutical University Nanjing China

**Keywords:** multifunctional hydrogels, regenerative medicine, tissue engineering, controlled drug delivery, rational design, clinical translation

## Abstract

Multifunctional hydrogels have emerged as advanced biomaterial platforms capable of integrating structural support, therapeutic delivery, and biological regulation to address the complex requirements of regenerative medicine. Their unique ability to mimic native extracellular matrix environments through tunable biochemical and biophysical properties has enabled broad applications in tissue repair and regeneration. This review provides a comprehensive overview of the rational design principles, fabrication strategies, and biomedical applications of multifunctional hydrogels. The fundamental relationships between polymer composition, crosslinking mechanisms, network architecture, and biological performance were firstly discussed, highlighting how these parameters regulate mechanical properties, degradation behavior, and cellular interactions. Subsequently, representative hydrogel systems, including natural polymers, synthetic polymers, and hybrid composites, are critically compared regarding their advantages, limitations, and suitability for different regenerative scenarios. We further examine functional strategies such as controlled therapeutic delivery, immunomodulation, cell encapsulation, and stimuli‐responsive regulation, with emphasis on their mechanisms and translational potential. Applications in wound healing, bone and cartilage regeneration, neural repair, and cardiovascular tissue engineering are discussed alongside current clinical challenges. Finally, we highlight key barriers including scalability, standardization, regulatory considerations, and future opportunities toward clinically translatable multifunctional hydrogel technologies.

## Graphical abstract

Multifunctional hydrogels represent a paradigm shift in regenerative medicine, integrating rational material design with bioactive functionality to address complex tissue repair challenges. This review systematically examines the molecular engineering, structure–function relationships, and translational landscape of advanced hydrogel systems, spanning natural biopolymers, synthetic polymers, and hybrid networks. By elucidating universal design principles, including dynamic crosslinking, stimuli‐responsiveness, and multitherapeutic cargo delivery, we provide a comprehensive framework for developing next‐generation regenerative therapies. The convergence of material science, biology, and clinical translation positions multifunctional hydrogels as transformative platforms for tissue engineering and precision medicine.

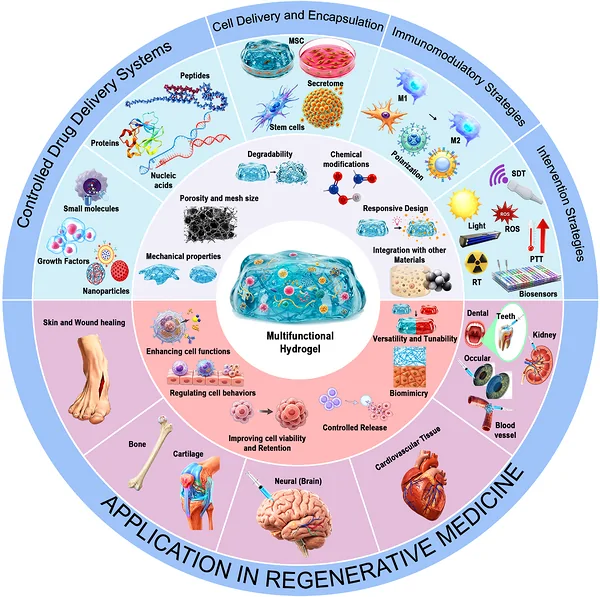

## 1 Introduction

Since the pioneering development of poly(2‐hydroxyethyl methacrylate) hydrogels by Wichterle and Lím in the 1960s for contact lens applications, hydrogels have become fundamental components of modern biomedical engineering due to their high‐water content, tissue‐like properties, and excellent biocompatibility [1]. Early biomedical applications primarily focused on passive functions, such as lubrication, wound coverage, and controlled drug diffusion [2]. However, advances in polymer chemistry, materials engineering, and regenerative biology have progressively transformed hydrogels from inert supports. Hydrogels have evolved from simple water‐swollen polymer networks into sophisticated biomaterial platforms capable of regulating complex biological processes.

The emergence of controlled drug delivery in the 1970s and 1980s represented a major milestone in hydrogel development. Their hydrated three‐dimensional (3D) networks provided a favorable reservoir for therapeutic molecules, enabling localized and sustained release while reducing systemic toxicity [3]. During this period, natural polymer‐based hydrogels, including collagen, alginate, and chitosan systems, further expanded the biomedical potential of hydrogels by introducing intrinsic biological signals that promote cell adhesion, migration, and tissue integration [4]. These advances established the foundation for the development of biomimetic materials capable of bridging the gap between synthetic engineering and biological regeneration. The field experienced a further paradigm shift with the emergence of tissue engineering and regenerative medicine. Increasing evidence demonstrated that cells respond not only to biochemical signals but also to the physical characteristics of their surrounding matrix, including stiffness, viscoelasticity, architecture, and degradation behavior [5]. Consequently, hydrogel design has moved beyond chemical composition toward precise regulation of material properties to control cellular fate and tissue formation. The development of photo‐crosslinkable hydrogels, injectable systems, and advanced fabrication approaches such as 3D bioprinting has further enabled spatial and temporal control over hydrogel formation, facilitating the construction of complex regenerative microenvironments [6].

Despite these advances, tissue regeneration remains a highly complex process involving coordinated interactions among immune responses, extracellular matrix (ECM) remodeling, vascularization, and cellular differentiation. Conventional single‐function hydrogels often fail to address the multifactorial nature of tissue injury because they primarily provide structural support or delivery of a single therapeutic component. In contrast, native ECM functions as a multifunctional biological framework that simultaneously provides mechanical support, regulates biochemical signaling, stores growth factors, modulates immune activity, and directs cellular organization [7]. Therefore, reproducing the dynamic and integrated functions of ECM has become a central objective in the development of next‐generation hydrogel systems.

Multifunctional hydrogels have emerged in response to this challenge by integrating multiple biological and physicochemical functions within a single platform. Through rational molecular design, these systems can combine mechanical reinforcement, controlled therapeutic delivery, immunomodulation, antimicrobial activity, antioxidant regulation, and cellular guidance (Figure 1). Such integration is particularly valuable for complex pathological conditions where multiple biological barriers coexist. For example, diabetic wounds exhibit persistent inflammation, excessive oxidative stress, impaired angiogenesis, and abnormal ECM remodeling, requiring simultaneous regulation of multiple pathways rather than a single therapeutic intervention [8]. Multifunctional hydrogels can address these challenges by providing adaptive mechanical support, delivering bioactive molecules, regulating immune responses, and promoting vascularized tissue regeneration within a localized microenvironment.

**FIGURE 1:**
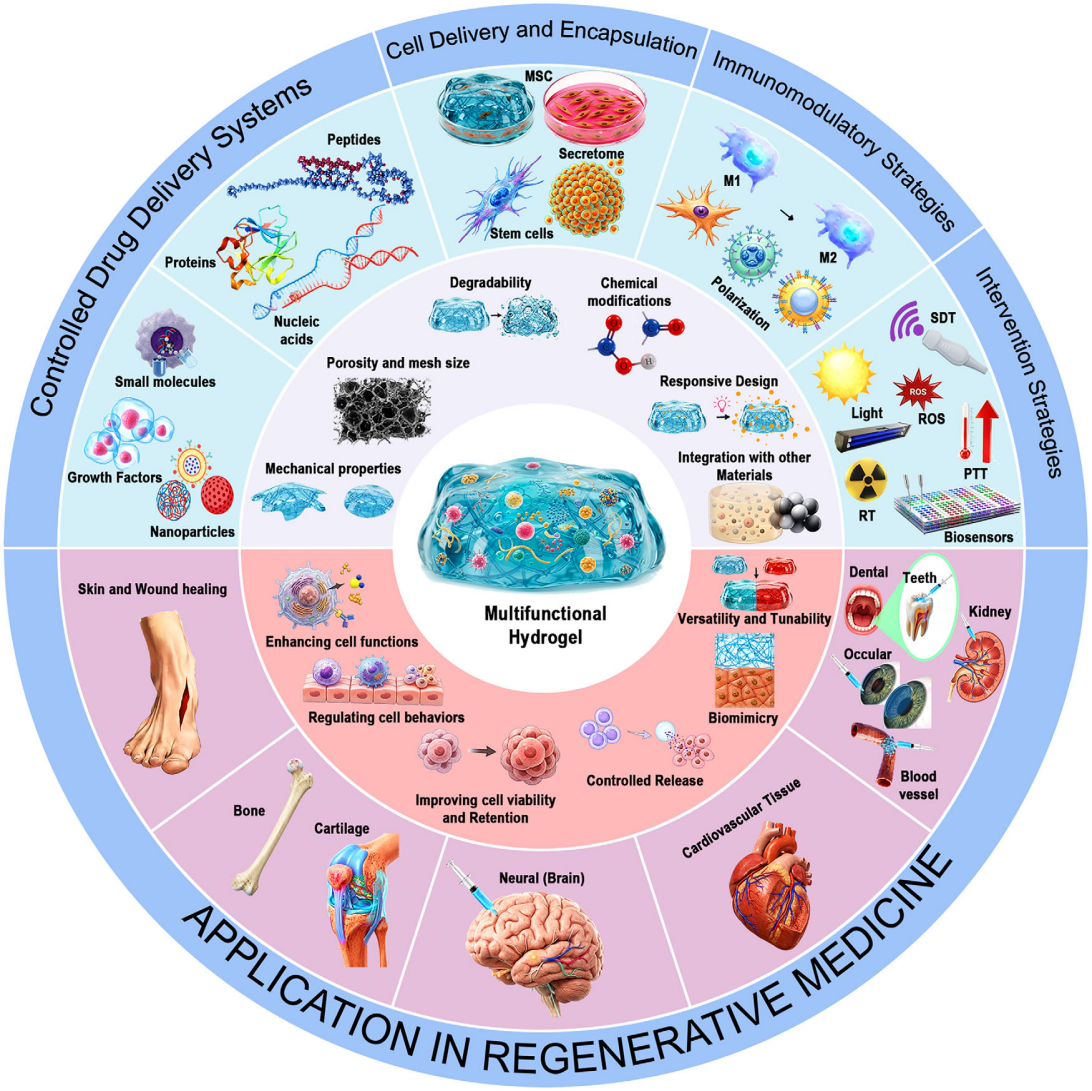
Overview of multifunctional hydrogel platforms for regenerative medicine. The central hydrogel core is characterized by its tunable physicochemical properties (mechanical, porosity, degradability, chemical modifications, responsiveness, and material integration), which enable four core therapeutic strategies: drug delivery, cell encapsulation, immunomodulation, and external‐stimulus intervention. These combined capabilities drive a wide range of applications, including skin, bone, cartilage, neural, cardiovascular, dental, ocular, and kidney tissue regeneration.

The therapeutic advantage of multifunctionality is not simply the addition of multiple components but the establishment of synergistic interactions between material properties and biological processes. For instance, immunomodulatory hydrogels that promote macrophage polarization toward a regenerative phenotype can enhance angiogenesis and tissue remodeling, while controlled release of vascular endothelial growth factor (VEGF) can further accelerate vascular maturation [9]. Similarly, the combination of appropriate mechanical cues and biochemical signals can improve stem cell survival, differentiation, and tissue integration [10]. These examples highlight the importance of designing multifunctional hydrogels based on fundamental structure–property–function relationships rather than empirical material combinations. Although significant progress has been achieved, several challenges continue to limit clinical translation. Many multifunctional hydrogel systems remain restricted to proof‐of‐concept studies due to difficulties associated with scalable manufacturing, batch‐to‐batch consistency, sterilization compatibility, long‐term safety evaluation, regulatory classification, and cost‐effectiveness. Furthermore, the complexity of multifunctional designs often complicates mechanistic understanding and standardized evaluation [11]. Therefore, establishing rational design principles and identifying universal strategies for translating multifunctional hydrogels from laboratory research to clinical applications remain critical priorities.

This review provides a comprehensive analysis of multifunctional hydrogels for regenerative medicine, highlighting the relationship between material design, biological functions, and translational potential. We discuss rational design strategies, including polymer selection, crosslinking, mechanical regulation, degradation control, and biointerface engineering, while comparing natural, synthetic, and hybrid hydrogel platforms. Emerging functionalities, regenerative applications, translational challenges, and future perspectives toward clinically deployable hydrogel technologies are also discussed to establish a framework for next‐generation tissue repair strategies.

## 2 Methodology Statement

This review was compiled through a systematic search of the Web of Science, PubMed, and Scopus databases for peer‐reviewed articles published between January 2020 and June 2026, using combinations of keywords including “multifunctional hydrogels,” “regenerative medicine,” “tissue engineering,” “stimuli‐responsive hydrogels,” “drug delivery,” “immunomodulation,” and “clinical translation.” Only English‐language articles reporting original research or comprehensive reviews on hydrogel‐based regenerative platforms were considered. For product‐specific regulatory information, official Food and Drug Administration (FDA) documentation and publicly accessible clinical trial registries (e.g., ClinicalTrials.gov) were consulted where available, with additional regulatory updates sourced from institutional announcements. Quantitative ranges for mechanical properties and degradation profiles were extracted from studies reporting standardized testing conditions; values should be interpreted as representative ranges rather than absolute material constants. Reference lists of selected reviews were screened for additional relevant studies. The selection of primary studies and the resolution of disagreements between authors were conducted through consensus discussion.

## 3 Fundamental Design Principles of Multifunctional Hydrogels

Multifunctional hydrogels integrate structural, biochemical, and biological functions to dynamically regulate the regenerative microenvironment. Their performance arises from the interplay between polymer chemistry, network architecture, and cell–material interactions. Rational design therefore requires the simultaneous optimization of mechanical properties, degradation behavior, bioactivity, and therapeutic functions, as improving one parameter often compromises another. Rather than relying on isolated material optimization, multifunctional hydrogel engineering follows an integrated structure–property–function strategy. Polymer chemistry provides the foundation of hydrogel design (Figure 2A), while the comparative advantages and limitations of natural, synthetic, and hybrid polymers are discussed in Section 3. The following sections focus on the key design principles (including crosslinking, mechanical regulation, degradation control, biological interactions, and multifunctional integration) that collectively determine regenerative performance and translational potential.

**FIGURE 2:**
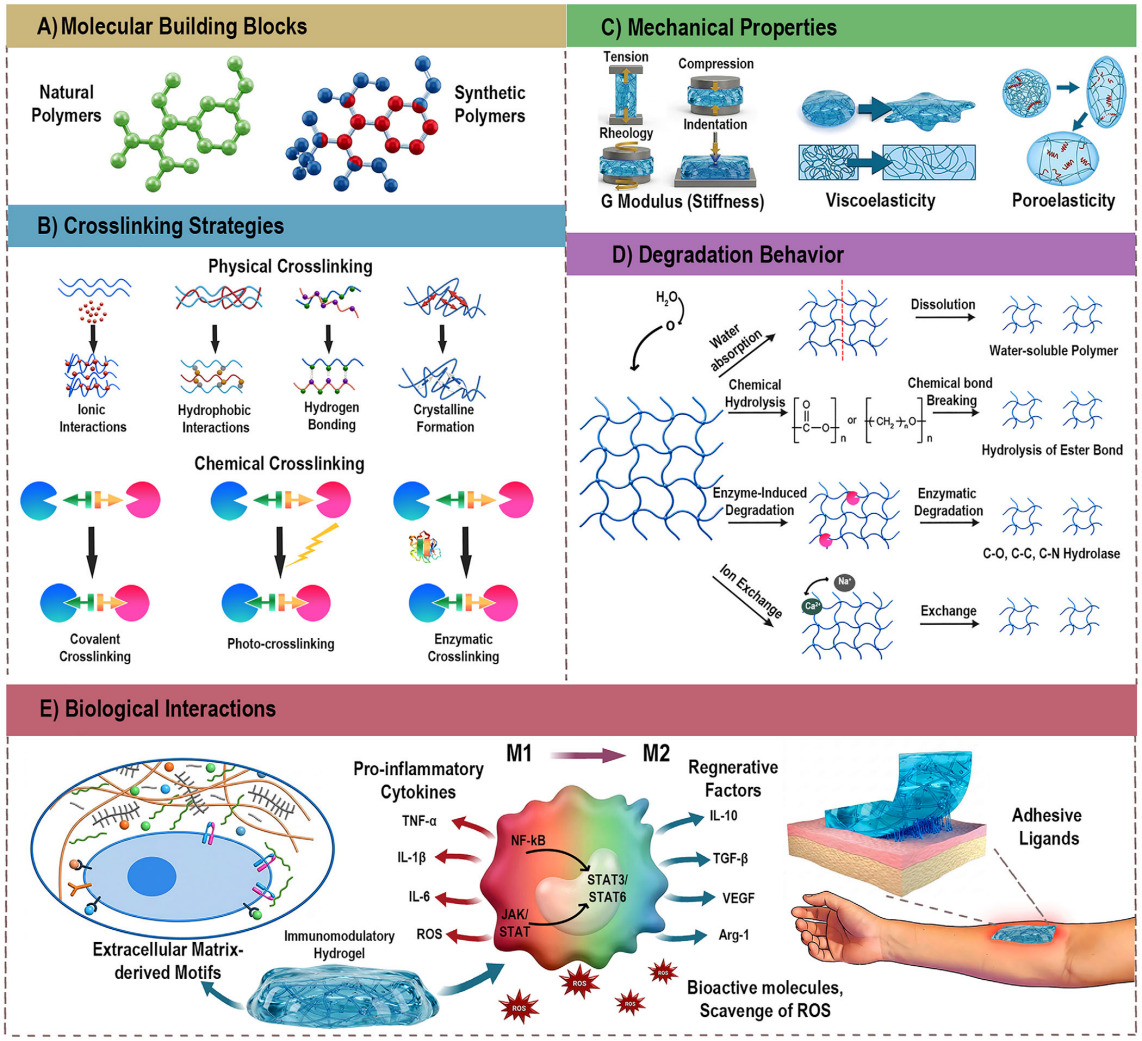
Design principles and properties of Multifunctional hydrogels. The figure details the hierarchical design of hydrogels, progressing from (A) molecular building blocks to (B) various physical and chemical crosslinking strategies. These properties determine the (C) mechanical characteristics and (D) degradation behavior (via hydrolysis, enzymatic action, or ion exchange). (E) The culmination of these physicochemical properties governs biological interactions, wherein ECM‐mimetic motifs and adhesive ligands guide immunomodulation. This ultimately facilitates macrophage polarization from a pro‐inflammatory (M1) to a pro‐regenerative (M2) state, alongside the scavenging of ROS, thereby creating a favorable microenvironment for tissue repair.

### 3.1 Crosslinking Strategies

Crosslinking determines hydrogel network architecture and directly governs gelation kinetics, mechanical strength, degradation behavior, and the encapsulation of cells or therapeutic cargos (Figure 2B). Crosslinking approaches are broadly classified into physical and chemical mechanisms, each offering distinct advantages and limitations depending on the intended biomedical application [12]. The following sections focus on the general principles of these crosslinking strategies, while polymer‐specific details are presented in Section 3 and Table 1.

**TABLE 1:** Comprehensive comparison of hydrogel systems for regenerative medicine.

Category	Hydrogel system	Crosslinking mechanism	Mechanical properties	Degradation profile	Bioactive cues	Advantages	Key limitations	Primary applications	Ref.
Photo‐crosslinkable natural polymers	Gelatin methacryloyl (GelMA)	UV/Vis photo‐polymerization (LAP 0.05–0.1 wt%, 10–60s)	G': 2–50 kPa (DS 60%–90%, 5%–20% w/v) E: 10–200 kPa	40%–60% mass loss in 4 weeks (MMP‐9); 80% in 8 weeks	RGD motifs (250–300 µmol/g), MMP‐sensitive (GPLGIAGQ); High adhesion	Cytocompatibility; tunable stiffness; Supports diverse cell types	G' < 50 kPa (load‐bearing unsuitable)	Wound healing, cartilage, bone, vascular, 3D bioprinting	[13, 14]
	Silk fibroin methacryloyl (SilMA/SF‐GMA)	Vis photo‐polymerization + β‐sheet physical (dual network)	G': 8–65 kPa (DS 15%–40%, 10%–20% w/v); β‐sheet 40%–60%	15%–25% mass loss in 4 weeks; 40%–60% 12 weeks (β‐sheet dependent)	RGD‐like motifs (FGGSS); limited antioxidant; Moderate adhesion	Highest mechanical; β‐sheet persistence; Injectable	Batch CV >20%; DS β‐sheet interference; high LAP requirement	Diabetic wounds, bone, nerve, cartilage, DLP 3D printing	[15, 16, 17, 18, 19, 20]
	Hyaluronic acid methacryloyl (HAMA)	UV/Vis photo‐polymerization	G': 0.4–8 kPa (DS 20%–50%, 1%–5% w/v)	50%–70% mass loss in 2 weeks (hyaluronidase); complete in 4 weeks	CD44 binding (Kd 10–100 µM); anti‐inflammatory; Moderate adhesion	CD44‐mediated migration; Native ECM hydration; Chondrogenic support	Rapid degradation; G' < 8 kPa;	Cartilage repair, wound healing, injectable fillers, osteoarthritis	[21, 22]
	Alginate methacrylate (AlgMA)	UV photo‐polymerization + ionic (Ca^2^^+^ 10–100 mM)	G': 5–60 kPa (DS 10%–40%, 2%–8% w/v)	10%–20% mass loss in 4 weeks; ion‐dependent	Bio‐inert (requires RGD); low adhesion	>98% cell viability (encapsulation); Low cost	No adhesion (requires RGD conjugation); limited mammalian degradation	Cell encapsulation, bioprinting, drug delivery	[22, 23]
	Chitosan methacrylate (ChiMA)	UV photo‐polymerization	G': 1–25 kPa (DS 20%–50%, 2%–6% w/v)	30%–50% mass loss in 4 weeks (lysozyme); 70% in 8 weeks	Antimicrobial (MIC 25–100 µg/mL); hemostatic; cationic (DD 75%–95%); moderate adhesion	antibacterial (*S. aureus*, *E. coli*); Hemostatic; Mucoadhesive	pH solubility (insoluble >pH 6.5); batch variability; brittle when dry	Wound healing, infection control, hemostasis	[22, 24]
Non‐photo‐crosslinkable natural proteins	Collagen (native)	Physical (fibrillogenesis, pH 7.4, 37°C); Chemical (EDC/NHS 1–10 mM)	G': 0.2–15 kPa (1–10 mg/mL); E: 1–100 kPa	40%–70% mass loss in 2 weeks (collagenase); 90% in 4 weeks	RGD motifs (GxOGER, GFOGER); native ECM; MMP‐sensitive; high adhesion	Native cell adhesion; Supports diverse cell types	Batch variability; pathogen risk (animal‐derived); weak mechanical (<15 kPa)	Wound healing, skin substitutes, bone, cartilage, hemostasis	[25, 26, 27]
	Fibrin (native)	Enzymatic (thrombin 1–10 U/mL)	G': 0.1–10 kPa (fibrinogen 10–50 mg/mL)	50%–80% mass loss @ 2 weeks (plasmin); complete 4 weeks	Cell‐adhesive (integrin αvβ3); growth factor binding (heparin, VEGF); High adhesion	Natural clotting; Growth factor sequestration (VEGF, FGF); Injectable	G' < 10 kPa; rapid degradation (2–4 weeks); expensive	Hemostasis, surgical sealants, stem cell delivery, wound healing	[28, 29]
	Hyaluronic acid (native)	Physical (hydrogen bonding); Chemical (BDDE, EDC/NHS)	G': 0.01–5 kPa (1%–5% w/v); E: 0.1–10 kPa	>80% mass loss @ 1 week (hyaluronidase); complete 2 weeks	CD44 binding (Kd 10–100 µM); anti‐inflammatory; hydration; moderate adhesion	Native ECM; CD44‐mediated signaling	G' < 5 kPa; degradatio*n* <1 week	Wound healing, osteoarthritis, dermal fillers, ophthalmic surgery	[30, 31]
	Alginate (native)	Ionic (Ca^2^^+^ 10–100 mM; Ba^2^^+^ for stronger)	G': 1–80 kPa (1%–5% w/v, Ca^2^^+^ 10–100 mM)	5%–15% mass loss in 4 weeks; ion‐dependent (Ca^2^^+^ leaching)	Bio‐inert (requires RGD conjugation); low adhesion	Gentle encapsulation (>95% viability); Low cost	No adhesion (requires RGD); ion‐dependent instability; no mammalian degradation	Cell encapsulation, drug delivery, wound dressings, 3D culture	[32, 33, 34, 35]
	Chitosan (native)	Physical (pH‐dependent); chemical (glutaraldehyde, genipin)	G': 0.1–15 kPa (1%–5% w/v, pH 4–6)	30%–60% mass loss @ 4 weeks (lysozyme); 80% in 8 weeks	Antimicrobial (MIC 25–100 µg/mL); hemostatic; cationic (DD 75%–95%); moderate adhesion	Antimicrobial; hemostatic	pH solubility (insoluble >pH 6.5); Batch variability; Brittle when dry	Wound healing, hemostasis, drug delivery, tissue engineering	[24, 36, 37, 38, 39]
Non‐photo‐crosslinkable synthetic polymers	Polyethylene glycol (PEG native)	Physical; chemical (multifunctional crosslinkers)	G': 5–150 kPa (MW 2–20 kDa, 5%–20% w/v)	Nondegradable (unless hydrolytic linkages introduced)	Bio‐inert (requires RGD, YIGSR, IKVAV); low adhesion	Excellent reproducibility; Bio‐inert	Bio‐inert; nondegradable; no intrinsic bioactivity	Drug delivery, wound healing, tissue engineering scaffolds, sealants	[40, 41]
	Polyvinyl alcohol (PVA native)	Physical (freeze‐thaw, −20°C to RT); chemical (glutaraldehyde)	G': 10–200 kPa (5%–15% w/v, freeze‐thaw cycles 1–5); E: 100–500 kPa	Slow hydrolytic (5%–15% mass loss/year); limited degradation	Bio‐inert (requires functionalization); low adhesion	High mechanical (G' >100 kPa); Low cost	Limited biodegradability; bio‐inert; requires surface modification	Tissue engineering, drug delivery, wound healing, cartilage repair	[42] (43)
Photo‐crosslinkable synthetic polymers	PEG diacrylate/dimethacrylate (PEG‐DA/PEG‐MA)	UV/vis photopolymerization (LAP 0.05–0.5 wt%, 5–120 s)	G': 5–250 kPa (MW 2–20 kDa, 5%–30% w/v)	Hydrolytic: 0%–100% @ 4–24 weeks (design‐dependent)	Bio‐inert (requires RGD, YIGSR, IKVAV); low adhesion	Precise control; mechanical range (5–250 kPa)	Requires RGD for adhesion; no intrinsic bioactivity; hydrolytic degradation only	3D culture, drug delivery, tissue engineering, sealants	[44, 45]
Stimuli‐responsive synthetic polymers	Poly(N‐isopropylacrylamide) (PNIPAAm)	Physical (hydrophobic at LCST 32°C); Chemical	G': 0.1–20 kPa (5%–15% w/v, temperature‐dependent)	Nondegradable	Bio‐inert (requires functionalization); low adhesion	Thermoreversible; injectable; cell sheet engineering	Nondegradable; weak mechanical (<20 kPa); requires functionalization	Injectable hydrogels, drug delivery, cell sheet engineering, 3D culture	[46] (47)
Polypeptide and self‐assembly	Self‐assembling peptide hydrogels	Noncovalent (β‐sheet, hydrophobic, ionic, RADA16‐I)	G': 0.1–15 kPa (0.5%–5% w/v); E: 0.5–20 kPa	Stimuli‐responsive (pH, ionic strength); tunable (1–12 weeks)	Bioactive sequences (RGD, IKVAV, YIGSR, laminin); High adhesion	RGD/I KVAV sequences encoded; Injectable via syringe	G' <15 kPa (weak); expensive; complex synthesis	Drug delivery, neural tissue engineering, hemostasis, wound healing	[48]
Composite and hybrid systems	Inorganic nanoparticle‐enhanced hydrogels	Physical (nanoparticle‐polymer interaction) + Chemical	G': 50–1000+ kPa (nanoparticle 5%–20% w/v); E: 100–5000 kPa	Variable (nanoparticle‐dependent; 4–24 weeks)	Osteoconductivity (HAp), mechanical reinforcement, bioactivity; variable adhesion	Mechanical enhancement (2–20×); osteoconductivity (HAp); Drug delivery capability	Complex formulation; Nanoparticle toxicity concern; Reproducibility challenges	Bone regeneration, cartilage repair, tissue engineering, orthopedic	[49, 50]
	Conductive hydrogels	Physical (polymer‐nanoparticle) + chemical (photo, ionic)	G': 0.5–200 kPa (variable); Conductivity: 0.01–10 S/m	Variable (4–24 weeks)	Electrical conductivity, electroactivity, cardiac/neural stimulation; variable adhesion	Conductivity (0.01–10 S/m); electrical stimulation; Bioelectronic integration	Complex synthesis; potential cytotoxicity; limited long‐term stability	Neural tissue engineering, cardiac repair, bioelectronics, sensors	[51, 52]
Emerging systems	DNA hydrogels	Physical (DNA hybridization; Watson‐Crick base pairing)	G': 0.1–15 kPa (1%–10% w/v)	Enzymatic (DNase); tunable (1‐12 weeks)	Programmable, sequence‐specific, bioactive; low adhesion	Programmable structure; sequence‐specific recognition; responsive (pH, temperature)	Expensive; complex synthesis; limited mechanical properties	Drug delivery, biosensors, cell culture, tissue engineering	[53, 54]

*Note*: Mechanical properties, degradation profiles, and bioactivity values are representative ranges compiled from the cited literature and may vary with polymer molecular weight, concentration, crosslinking density, and testing conditions. Unless otherwise specified, values are based on standard formulations and testing protocols.

Abbreviations: AlgMA, alginate methacrylate; ChiMA, chitosan methacrylate; CV, coefficient of variation; DD, degree of deacetylation; DLP, digital light processing; DS, degree of substitution; E, elastic modulus; EDC, 1‐ethyl‐3‐(3‐dimethylaminopropyl)carbodiimide; FGGSS, fibroin‐derived peptide sequence; G', storage modulus; GelMA, gelatin methacryloyl; GPLGIAGQ, MMP‐sensitive peptide sequence; GxOGER, collagen‐derived integrin‐binding motif; HA, hyaluronic acid; HAMA, hyaluronic acid methacryloyl; HAp, hydroxyapatite; IKVAV, laminin‐derived peptide sequence; LAP, lithium phenyl‐2,4,6‐trimethylbenzoylphosphinate; LCST, Lower critical solution temperature; MIC, Minimum inhibitory concentration; MMP, matrix metalloproteinase; MW, molecular weight; NHS, N‐hydroxysuccinimide; PAM, polyacrylamide; PEG, polyethylene glycol; PEGDA, polyethylene glycol diacrylate; PNIPAAm, poly(N‐isopropylacrylamide); PVA, polyvinyl alcohol; RADA16‐I, self‐assembling peptide sequence; RGD, arginine‐glycine‐aspartic acid; RHAMM, receptor for hyaluronic acid‐mediated motility; SF, silk fibroin; SF‐GMA, silk fibroin‐glycidyl methacrylate; SilMA, silk fibroin methacryloyl; UV, ultraviolet; VEGF, vascular endothelial growth factor; Vis, visible light; w/v, weight per volume; YIGSR, laminin‐derived peptide sequence.

#### 3.1.1 Physical Crosslinking

Physical crosslinking relies on reversible non‐covalent interactions, providing injectable, self‐healing, and stimuli‐responsive hydrogels. However, their reversible nature generally limits long‐term mechanical stability compared with covalently crosslinked systems [55]. Hydrogen bonding is widely employed in polymers such as polyvinyl alcohol, where repeated freeze–thaw cycles induce physically crosslinked networks with improved mechanical integrity [56]. Ionic crosslinking, commonly achieved through multivalent cation‐mediated interactions, enables rapid gelation under physiological conditions and is particularly suitable for cell encapsulation, although ion exchange often results in poor mechanical stability and uncontrolled degradation [57]. Hydrophobic interactions drive the self‐assembly of amphiphilic polymers, exemplified by thermoresponsive systems that undergo temperature‐dependent sol–gel transition and are particularly attractive for injectable therapies despite relatively weak mechanical properties [58, 59]. Other reversible interactions, including π–π stacking and host–guest inclusion complexes such as cyclodextrin–adamantane systems, further enhance network stability while preserving dynamic responsiveness and enabling modular hydrogel assembly with tunable mechanical and stimuli‐responsive properties [60, 61].

#### 3.1.2 Chemical Crosslinking

Chemical crosslinking forms covalent networks with superior mechanical stability, prolonged structural integrity, and more predictable degradation profiles [62]. Nevertheless, reaction conditions must be carefully optimized to preserve the viability of encapsulated cells and bioactive molecules; which including reactants and by‐products that are difficult to eliminate completely.

Free‐radical polymerization remains the most widely used approach, particularly for methacrylated biomaterials such as gelatin methacryloyl, hyaluronic acid methacryloyl, and silk fibroin‐glycidyl methacrylate [63, 64]. Photo‐initiated polymerization enables rapid in situ gelation with excellent spatial and temporal control, making it highly compatible with bioprinting and minimally invasive applications [65, 66]. However, photoinitiators and reactive oxygen species may compromise cellular function if exposure parameters (photoinitiator concentration typically 0.05–0.5 wt%, light intensity 5–50 mW/cm^2^, irradiation wavelength 365–405 nm, and exposure duration 5–120 s) are not optimized.

Bioorthogonal click chemistry has emerged as an attractive alternative because of its high specificity and cytocompatibility. Copper‐catalyzed azide–alkyne cycloaddition provides efficient crosslinking but is limited by catalyst toxicity, whereas strain‐promoted azide–alkyne cycloaddition eliminates metal catalysts while maintaining excellent biocompatibility [67, 68]. Thiol–ene, thiol–yne, and Michael addition reactions further enable rapid gelation under physiological conditions and are widely applied in cell‐laden hydrogels [69, 70]. Dynamic covalent chemistries, including Schiff‐base reactions, generate reversible networks with self‐healing capability, although aldehyde toxicity requires careful optimization [71, 72]. Among these bio‐click chemistries, enzyme‐mediated crosslinking, such as transglutaminase‐catalyzed reactions, offers exceptional cytocompatibility under physiological conditions, although it remains constrained by enzyme specificity and production cost [73].

Beyond providing structural integrity, crosslinking strategies have evolved into functional design tools that regulate hydrogel adaptability. Static covalent networks offer mechanical stability but may poorly mimic the dynamic remodeling behavior of native ECM. Conversely, reversible and dynamic crosslinking mechanisms introduce self‐healing capability, stress relaxation, and environmental responsiveness. Therefore, future multifunctional hydrogels will likely incorporate complementary crosslinking approaches to achieve both long‐term stability and biological adaptability.

### 3.2 Mechanical Property Regulation

Mechanical properties (including stiffness, viscoelasticity, toughness, elasticity, and porosity) critically influence hydrogel performance by regulating cell behavior, therapeutic delivery, and scaffold stability (Figure 2C). Therefore, matching hydrogel mechanics to native tissue characteristics is a fundamental design principle in regenerative medicine.

Hydrogel stiffness, typically represented by the storage modulus (G′), regulates mechanotransduction and influences stem cell differentiation, immune activation, angiogenesis, and fibroblast function [74, 75]. Stiffness is commonly adjusted by varying polymer concentration, crosslinking density, or degree of functionalization. However, these modifications simultaneously affect mesh size and degradation behavior. Alternatively, reinforcing nanomaterials, including hydroxyapatite nanoparticles, nanoclays, and carbon nanomaterials, enable independent enhancement of mechanical strength while preserving network architecture [76, 77].

Beyond stiffness, native tissues exhibit pronounced viscoelasticity, characterized by stress relaxation, creep, and hysteresis. Dynamic hydrogels capable of stress relaxation more closely mimic physiological ECM behavior and have been shown to enhance cell spreading, proliferation, and lineage‐specific differentiation [78, 79, 80]. For load‐bearing applications, hydrogel toughness is equally important. Double‐network hydrogels combine a sacrificial brittle network with a flexible elastic network, achieving fracture resistance approaching that of native cartilage [81, 82]. In addition, poroelastic properties regulate fluid transport, nutrient diffusion, and mechanical response, making them particularly relevant for cartilage and intervertebral disc regeneration [83].

Mechanical properties should not be considered as isolated parameters but as biological regulators. Native tissues exhibit complex mechanical behaviors involving stiffness, viscoelasticity, and stress relaxation rather than simple elastic responses. Accordingly, next‐generation hydrogels should move beyond stiffness matching toward dynamic mechanical regulation capable of guiding cell fate, immune responses, and tissue remodeling throughout different regeneration stages.

### 3.3 Degradation Behavior

Hydrogel degradation determines therapeutic release profiles and tissue remodeling. Ideally, degradation kinetics should coincide with the regenerative timeline, providing temporary support while allowing progressive tissue ingrowth [84]. Hydrolytic degradation occurs through water‐mediated cleavage of hydrolytically labile bonds and offers predictable degradation independent of cellular activity (Figure 2D) [85]. Although suitable for controlled therapeutic release, hydrolytic degradation cannot adapt to dynamic tissue remodeling. Conversely, enzymatic degradation enables cell‐responsive scaffold remodeling through protease‐sensitive sequences incorporated into the hydrogel network. Matrix metalloproteinase (MMP)‐responsive hydrogels represent a prominent example, where scaffold degradation directly correlates with local cellular activity and ECM remodeling [86, 87]. These systems incorporate peptide sequences cleaved by cell‐secreted matrix metalloproteinases, enabling degradation that is directly coupled to cellular activity and tissue remodeling demands. Furthermore, degradation products should remain biocompatible and ideally support regenerative processes without provoking adverse inflammatory responses [88].

An ideal regenerative hydrogel should not simply resist degradation but should undergo controlled remodeling synchronized with tissue formation. Excessively slow degradation may hinder cellular infiltration and matrix replacement, whereas rapid degradation can result in premature loss of structural support. Therefore, programmable degradation represents a critical component of multifunctional design, allowing hydrogels to evolve with the changing biological requirements of regenerating tissues.

### 3.4 Biological Interactions

The therapeutic efficacy of multifunctional hydrogels depends on their ability to regulate cell–material interactions and biological responses. Cell adhesion is primarily mediated by ECM‐derived motifs (Figure 2E), with the RGD sequence widely used to promote cell attachment, spreading, and survival [89, 90]. Other bioactive peptides, including IKVAV, YIGSR, REDV, and GFOGER, provide tissue‐specific signals that further enhance regenerative outcomes [91]. In contrast, bioinert polymers such as polyethylene glycol (PEG) require functional modification with adhesive ligands to improve cellular integration while minimizing nonspecific interactions [92].

Beyond cellular adhesion, immune regulation has become a critical design consideration in regenerative hydrogel engineering. Material properties, including surface chemistry, charge, hydrophobicity, and degradation products, influence macrophage polarization and inflammatory responses [10]. While excessive M1 macrophage activation contributes to chronic inflammation and fibrosis, promoting regenerative M2 polarization can enhance tissue repair [93]. Accordingly, multifunctional hydrogels increasingly incorporate immunomodulatory molecules, cytokines, or bioactive factors to actively regulate immune responses and establish pro‐regenerative microenvironments [94, 95]. Physical and electrical cues transduced by hydrogels provide an additional layer of biological regulation beyond biochemical signaling. Mechanotransduction (governed by matrix stiffness, porosity, and viscoelasticity) directs stem cell migration, fate specification, and lineage commitment through pathways such as YAP/TAZ, while electroactive hydrogels incorporating conductive polymers or nanomaterials facilitate electromechanical and electrochemical signal transduction, regulating excitable cell behavior including neuronal differentiation, cardiomyocyte synchronization, and guided cell migration [94, 95].

These advances have shifted hydrogels from passive ECM mimics toward active biological regulators. By integrating cell‐instructive signals and immune‐modulating functions, next‐generation hydrogels can coordinate multiple regenerative processes and overcome limitations associated with conventional tissue engineering strategies.

### 3.5 Multifunctionality as a Strategy to Address Complex Regenerative Challenges

Tissue regeneration involves highly coordinated biological events, including inflammation resolution, angiogenesis, matrix deposition, and functional remodeling. Because these processes occur simultaneously and interact with each other, single‐function materials often provide insufficient therapeutic effects. Multifunctional hydrogels overcome this limitation by integrating complementary functions within a unified platform [96]. For example, combining immunomodulation with angiogenic factor delivery can promote vascular regeneration while controlling excessive inflammation [97]. Similarly, mechanical reinforcement combined with biochemical signaling can enhance stem cell survival and tissue integration [98]. Therefore, multifunctionality should be viewed as a strategy to address biological complexity rather than simply increasing material complexity.

## 4 Classification and Comparative Analysis of Hydrogel Systems

The selection of polymer chemistry is the foundational decision in hydrogel design, establishing the framework within which all subsequent functional properties are achieved. This selection must balance biocompatibility, processability, mechanical properties, degradation behavior, and available chemical handles for functionalization. The following sections classify and compare the major hydrogel systems according to their polymer origin, crosslinking mechanisms, and biomedical applications, with comprehensive quantitative data summarized in Table 1.

### 4.1 Naturally‐Derived Hydrogel Systems (Biomimetic Design)

Biomimetic design prioritizes biological functionality over engineering precision, seeking to replicate aspects of the native ECM through the use of naturally‐derived polymers (Figure 3A).

**FIGURE 3:**
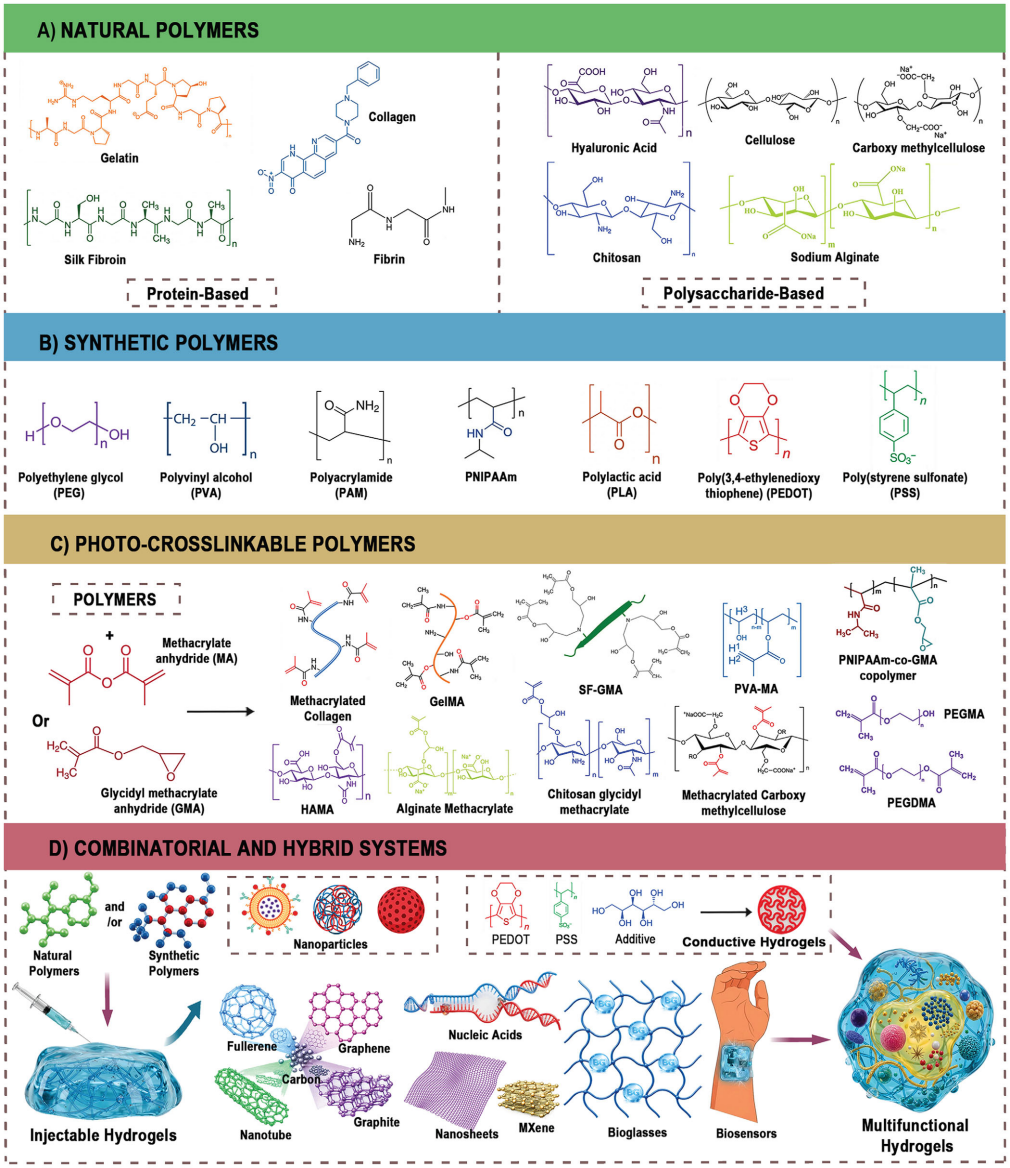
Classification of polymer systems for multifunctional hydrogel fabrication. (A) Natural polymers, including protein‐ and polysaccharide‐based structures. (B) Common synthetic polymers with precisely defined backbones. (C) Photocrosslinkable derivatives obtained through methacrylation or glycidyl methacrylation of natural and synthetic polymers. (D) Combinatorial and hybrid systems incorporating nanomaterials (e.g., nanoparticles, carbon allotropes, MXenes), conductive polymers, and biomolecules, which can be leveraged to generate injectable hydrogels, bio‐glues, and biosensors.

#### 4.1.1 Protein‐Based Hydrogels

Naturally derived polymers possess intrinsic bioactivity and structural similarity to the native ECM, making them attractive for regenerative medicine.

Fibrin hydrogels, formed through thrombin‐mediated polymerization of fibrinogen, exploit the body's natural clotting mechanism to create biodegradable, cell‐adhesive networks [28, 29]. The inherent binding sites for growth factors and cell adhesion receptors make fibrin hydrogels attractive for regenerative applications, while the rapid degradation and mechanical weakness limit load‐bearing applications. Fibrin hydrogels have found clinical use as hemostatic agents and surgical sealants, with emerging applications in cell delivery and drug release.

Collagen promotes cell adhesion and matrix remodeling through abundant integrin‐binding motifs but exhibits limited mechanical strength and batch variability [25]. Collagen hydrogels, typically derived from type I collagen extracted from bovine or porcine sources, preserve the native triple‐helical structure and cell‐adhesive RGD motifs that support diverse cell types [26, 27]. Their fibrillar nature provides a topography that guides cell alignment and tissue organization, while the hydrogel's innate susceptibility to enzymatic degradation enables cell‐mediated remodeling [99]. However, these hydrogels exhibit weak mechanical properties, rapid contraction by encapsulated cells, and limitations in reproducibility and pathogen safety. Despite the challenges, collagen hydrogels remain widely used in research and have found clinical applications in wound healing and soft tissue repair.

Gelatin, a denatured collagen derivative, retains bioactive sequences while offering improved solubility and processability [100]. Gelatin hydrogels can be physically crosslinked at reduced temperatures (typically 4–20°C, with sol–gel transition occurring around 22–33°C and complete gelation at ∼17°C) or chemically crosslinked with glutaraldehyde, genipin, or transglutaminase (typically performed at 37°C or room temperature, though enzymatic crosslinking can also be conducted above and below the physical gelation temperature). However, simple gelatin hydrogels suffer from poor mechanical properties and rapid degradation, motivating extensive chemical modification [101, 102]. Gelatin methacryloyl (GelMA) represents the most successful gelatin derivative, enabling photo‐crosslinked hydrogel formation with tunable mechanical properties and controlled degradation [13]. The combination of cell‐adhesive properties, facile synthesis, and photo‐crosslinkability has made GelMA a versatile platform for numerous tissue engineering applications [14].

Silk fibroin (SF) combines superior mechanical strength, tunable β‐sheet‐mediated degradation, excellent cytocompatibility, and versatile processing, making it a promising platform for multifunctional hydrogel engineering [103]. SF hydrogels offer unique advantages in mechanical properties, processing options, and stability [104]. SF self‐assembles through β‐sheet formation, providing physical crosslinking that yields mechanically robust hydrogels without chemical modification [105, 106]. The extensive hydrophobic domains and high glycine‐alanine content contribute to silk's exceptional mechanical strength and resistance to degradation. Methacrylation of silk fibroin (SF‐GMA) enables photo‐crosslinking while preserving β‐sheet formation, creating hydrogels with dual physical and chemical crosslinking and exceptional mechanical properties [15, 16]. These hydrogels have demonstrated biocompatibility and support for diverse cell types in vitro and in vivo, with applications spanning bone, cartilage, nerve, and skin regeneration [17, 18, 19, 20].

#### 4.1.2 Polysaccharide‐Based Hydrogels

Hyaluronic acid (HA) regulates cell signaling through CD44 and RHAMM receptors and provides excellent hydration, although rapid enzymatic degradation necessitates chemical modification [30, 107]. HA hydrogels exploit the exceptional hydration capacity and biological signaling properties of this ubiquitous glycosaminoglycan [30, 31]. HA's interaction with CD44 receptors influences cell migration, proliferation, and differentiation, while its native role in embryogenesis and wound healing provides biological rationale for regenerative applications [108]. Chemical modification of HA hydroxyl and carboxyl groups enables diverse crosslinking strategies, with methacrylation (HAMA) enabling photo‐crosslinked hydrogels with tunable properties [21]. However, HA hydrogels exhibit rapid enzymatic degradation through hyaluronidases, motivating strategies for degradation control through crosslinking density and incorporation of inhibitors.

Chitosan exhibits antimicrobial, hemostatic, and cationic properties but has poor physiological solubility [36]. Chitosan hydrogels, derived from chitin deacetylation, offer antimicrobial activity, hemostatic properties, and biocompatibility that make them attractive for wound healing applications [37]. The cationic nature of chitosan enables ionic interactions with negatively charged cell membranes and therapeutic payloads, providing opportunities for drug delivery and cell adhesion [38]. Chitosan hydrogels can be physically crosslinked through pH‐sensitive interactions or chemically crosslinked with glutaraldehyde, genipin, or other agents [24, 39]. However, the limited solubility at physiological pH and batch variability constrain broader applications.

Alginate enables rapid ionic gelation with excellent biocompatibility but lacks intrinsic mammalian cell‐adhesive motifs and degrades slowly in vivo [32, 33]. Alginate hydrogels, crosslinked ionically with divalent cations, provide exceptionally gentle encapsulation conditions that have made alginate the most widely used polymer for cell transplantation [34, 35]. The inert nature of alginate minimizes inflammatory responses while providing a protective immuno‐isolation barrier for encapsulated cells. However, alginate lacks cell‐adhesion motifs and degradation mechanisms in mammalian systems, requiring chemical modification or composite strategies for most tissue engineering applications [109]. The inability of mammalian cells to degrade alginate limits its use as a cell‐instructive scaffold, though alginate hydrogels have been clinically approved as cell transplantation vehicles.

Cellulose derivatives, including hydroxypropyl methylcellulose, carboxymethyl cellulose, and methylcellulose, offer biocompatibility, low cost, and extensive regulatory history in pharmaceutical applications [110]. Cellulose hydrogels can be formed through physical crosslinking at elevated temperatures or chemical crosslinking [111, 112]. While cellulose lacks intrinsic biological signaling, its exceptional water retention and rheological properties make it valuable for wound healing and drug‐delivery formulations [113].

### 4.2 Synthetic Hydrogel Systems (Reductionist Design)

Reductionist design prioritizes engineering precision over biological complexity, employing well‐defined synthetic polymers that can be precisely characterized and reproducibly manufactured (Figure 3B).

#### 4.2.1 Polyethylene Glycol‐Based Hydrogels

Synthetic polymers offer excellent reproducibility, scalable manufacturing, and precise control over physicochemical properties. Polyethylene glycol (PEG) is widely used because of its biocompatibility, resistance to protein adsorption, and versatile functionalization, although its bioinert nature requires incorporation of adhesive ligands [40]. PEG hydrogels represent the most extensively studied synthetic hydrogel platform, benefiting from exceptional biocompatibility, protein‐repellent properties, and well‐established chemistry [41]. PEG's ability to resist non‐specific protein adsorption reduces inflammatory responses and enables precise biological signal presentation through intentional functionalization. The versatility of PEG synthesis enables diverse architectures, including linear, branched, and multiarm PEG, with terminal functional groups including acrylates, methacrylates, maleimides, and vinyl sulfones, enabling various crosslinking chemistries [114]. These hydrogels formed through photocrosslinking of PEG diacrylate or PEG dimethacrylate provide precise spatiotemporal control and compatibility with additive manufacturing [115]. Degradation can be incorporated through hydrolytically or enzymatically labile crosslinks, enabling timed network breakdown. However, the bio‐inert nature of PEG requires incorporation of cell‐adhesive and enzyme‐sensitive motifs for most regenerative applications, increasing system complexity.

#### 4.2.2 Polyvinyl Alcohol Hydrogels

Polyvinyl alcohol (PVA) provides high mechanical stability and film‐forming capability but lacks biological recognition sites [43]. PVA hydrogels offer robust mechanical properties, excellent water solubility, and an established safety profile in pharmaceutical applications [43]. Physical crosslinking through freeze‐thaw cycling creates mechanically robust networks without chemical crosslinkers, while chemical crosslinking with glutaraldehyde or other agents provides additional tunability. PVA hydrogels have been explored for cartilage repair, wound healing, and drug delivery applications. However, PVA's lack of biological recognition and limited degradability constrain its use in regenerative applications requiring cell infiltration and tissue remodeling [43].

#### 4.2.3 Polyacrylamide and Polypeptide Hydrogels

Polyacrylamide (PAM) enables precise mechanical tuning for mechanobiology studies but is generally unsuitable for implantation due to limited biodegradability [116, 117]. PAM hydrogels, while limited in direct biomedical applications due to biocompatibility concerns, have proven invaluable as model systems for studying cell–material interactions [118]. The precisely tunable mechanical properties, optical transparency, and compatibility with surface functionalization have made PAM hydrogels essential for mechanobiology studies establishing fundamental principles of cell response to substrate stiffness.

Polypeptide hydrogels, synthesized through recombinant or synthetic approaches, offer precise sequence control and monodispersity not achievable with natural polymers. Elastin‐like polypeptides, silk‐like polypeptides, and other engineered proteins can be designed to incorporate specific cell‐adhesive, enzyme‐sensitive, and stimuli‐responsive sequences, enabling highly sophisticated material design [119]. Poly(lactic‐co‐glycolic acid) (PLGA) is frequently incorporated into composite hydrogels to improve mechanical properties and provide controlled therapeutic release with established clinical safety [120, 121]. However, the cost and complexity of polypeptide synthesis limit current applications.

#### 4.2.4 Stimuli‐Responsive Synthetic Systems

Thermoresponsive hydrogels, exemplified by poly(N‐isopropylacrylamide) (PNIPAAm), undergo sol–gel transitions at specific temperatures, enabling injectable formulations that gel at body temperature [46]. pH‐responsive hydrogels, incorporating acidic or basic groups, release payloads in response to acidic wound microenvironments [122]. Ionic‐responsive hydrogels respond to changes in ionic strength or specific ions, with alginate‐Ca^2^^+^ systems being a classic example exploited in 4D printing and on‐demand drug delivery. Photoresponsive hydrogels enable spatiotemporally controlled drug release and network remodeling. The therapeutic strategies enabled by these systems, including photothermal, photodynamic, sonodynamic, and radiotherapy‐enhancing approaches, are discussed in detail in Section 4.4.

### 4.3 Photo‐Crosslinkable Hydrogel Systems

These hydrogels have emerged as a particularly versatile platform, enabling precise spatiotemporal control over gelation, mechanical property tuning, and compatibility with advanced fabrication techniques such as digital light processing (DLP), stereolithography (SLA), two‐photon polymerization, and microfluidics [123]. The light‐triggered polymerization of methacrylate groups, acrylates, or other unsaturated moieties enables rapid gelation under mild conditions (Figure 3C) [124].

Photoinitiator selection critically influences hydrogel properties and biocompatibility. Type I photoinitiators, including Irgacure 2959 and LAP, undergo unimolecular cleavage upon light absorption to generate reactive radicals [125]. Type II photoinitiators require a co‐initiator for radical generation. LAP offers particular advantages due to its high‐water solubility, absorption in the visible spectrum (365–405 nm), and cytocompatibility, making it widely used for cell‐laden hydrogel formation [126]. Irgacure 2959 is commonly employed in ultraviolet (UV)‐based tissue engineering scaffolds, whereas eosin Y and riboflavin (Type II) serve in ophthalmic and soft tissue applications where mild crosslinking is required.

Photocrosslinkable natural polymers, including GelMA, HAMA, chitosan methacrylate, alginate methacrylate, and SF‐GMA, combine the biological advantages of natural polymers with photocrosslinking control [22, 23]. Synthetic photo‐crosslinkable polymers, including PEG diacrylate and PEG dimethacrylate, provide precise mechanical property control and compatibility with photolithographic patterning [44, 45]. The integration of photocrosslinking with additive manufacturing, including DLP and stereolithography, enables the fabrication of complex, patient‐specific constructs with spatially resolved mechanical and biochemical properties [127].

Their biological performance is influenced by photoinitiator concentration, light exposure parameters, and residual reactive species. Optimized protocols achieve high cell viability (>90%) while maintaining mechanical integrity [125]. However, photo‐crosslinking presents challenges for translation, including light attenuation in biological tissues, potential genotoxicity of UV light, and regulatory considerations for photopolymerized materials, which require demonstration of photoinitiator safety, residual monomer characterization, and light‐source validation.

### 4.4 Combinatorial and Hybrid Systems

Combinatorial and hybrid hydrogels integrate natural polymers, synthetic polymers, and functional additives to achieve synergistic biological and mechanical properties unattainable with single‐component systems (Figure 3D). Although this strategy enhances multifunctionality, it also increases formulation complexity, manufacturing challenges, and regulatory barriers.

#### 4.4.1 Natural‐Synthetic Composites

Natural‐synthetic composites combine the biological activity of natural polymers with the tunable physicochemical properties of synthetic materials, thereby overcoming many limitations associated with individual components. Representative systems, including collagen–HA, gelatin–chitosan, alginate–silk fibroin, GelMA–PEG, and alginate–PEG, exhibit improved mechanical stability, controlled degradation, and enhanced cell–material interactions compared with single‐polymer hydrogels [128, 129]. The incorporation of ECM components, such as fibronectin, laminin, and proteoglycans, further strengthens biomimetic signaling and supports tissue‐specific regeneration [130, 131, 132]. Importantly, adjusting the ratio between natural and synthetic polymers enables precise regulation of swelling behavior, mechanical properties, and biological functionality to meet the requirements of different regenerative applications.

#### 4.4.2 Nanoparticle‐Enhanced Hydrogels

Nanoparticle incorporation represents a versatile strategy to augment hydrogel functionality, enabling controlled drug release, mechanical reinforcement, and the introduction of bioactive or stimuli‐responsive properties [49]. Polymeric nanoparticles, liposomes, inorganic nanomaterials, and extracellular vesicles (EVs) can be integrated into hydrogel networks to protect therapeutic payloads, modulate release kinetics, and enable co‐delivery of multiple agents with distinct spatiotemporal profiles [49].

Beyond drug delivery, nanoparticles confer additional functionalities. Hydroxyapatite and calcium phosphate nanoparticles enhance osteoconductivity and mechanical reinforcement for bone regeneration [50]. Bioactive glass nanoparticles promote angiogenesis and osteogenesis through controlled ion release. Other nanomaterials introduce photothermal therapy, magnetic responsiveness, antimicrobial activity, or imaging capabilities [133, 134]. However, nanoparticle incorporation increases formulation complexity and raises challenges related to colloidal stability, manufacturing reproducibility, long‐term biosafety, and regulatory approval.

#### 4.4.3 Conductive Hydrogels

Conductive hydrogels integrate electrically conductive components, including poly(3,4‐ethylenedioxythiophene) polystyrene sulfonate (PEDOT:PSS), polyaniline, graphene, carbon nanotubes, or metal nanoparticles, within hydrogel networks to enable bioelectronic applications [51]. These systems combine the biocompatibility and hydration of hydrogels with electrical conductivity, making them attractive for neural tissue engineering, cardiac repair, and bioelectronic interfaces. Conductivity ranges from 0.01 to 10 S/m, depending on the conductive component and loading. These hydrogels support electrical stimulation that can enhance cell alignment, differentiation, and functional integration. In cardiac applications, conductive hydrogels improve synchronous contraction and electrical coupling between cardiomyocytes. In neural applications, they support neurite outgrowth and electrical signal propagation [52]. However, conductive hydrogels face challenges including complex synthesis, potential cytotoxicity of conductive components, and limited long‐term stability.

#### 4.4.4 Self‐Assembling and DNA Hydrogels

Self‐assembling peptide hydrogels form through noncovalent interactions including β‐sheet formation, hydrophobic interactions, and ionic complementarity. The RADA16‐I peptide, for example, self‐assembles into nanofibrous networks that support cell adhesion and tissue integration [48]. These systems offer injectable delivery and defined bioactive sequences but exhibit weak mechanical properties (G': 0.1–15 kPa) and high cost.

DNA hydrogels exploit the programmable nature of DNA hybridization to form hydrogels with sequence‐specific recognition and stimuli‐responsive behavior [53]. DNA hydrogels offer precise control over network architecture, responsiveness to molecular signals, and biocompatibility [54]. However, they face limitations in mechanical properties, cost, and complex synthesis. DNA hydrogels have been explored for drug delivery, biosensing, and cell culture applications.

### 4.5 Trade‐Offs in Material Selection

Selection of an appropriate hydrogel requires balancing competing material properties rather than optimizing a single parameter. Four primary trade‐offs govern material selection: bioactivity versus reproducibility, mechanical properties versus degradation, manufacturability versus functionality, and cost versus performance. Natural polymers provide excellent bioactivity but often suffer from limited reproducibility, whereas synthetic polymers offer superior engineering control at the expense of biological functionality. Hybrid systems partially overcome these limitations but increase manufacturing complexity. Similarly, enhanced mechanical strength frequently compromises degradation and tissue remodeling, while highly functional systems introduce challenges related to scalability, quality control, regulatory approval, and cost. Consequently, hydrogel selection should be application‐driven. GelMA, chitosan, and alginate are well suited for wound healing, whereas SilMA, PEG‐based, nanoparticle‐reinforced, and injectable hydrogels are preferred for mechanically demanding or minimally invasive regenerative applications (Table 1).

## 5 Integration of Bioactive functionalities

The therapeutic efficacy of multifunctional hydrogels depends not only on their structural and mechanical properties but also on their ability to deliver bioactive cues that actively regulate cellular behavior and tissue regeneration. This section examines the major strategies for integrating therapeutic functionalities within hydrogel platforms, including controlled drug delivery systems, cell encapsulation and delivery, immunomodulation, and stimuli‐responsive behaviors, while critically assessing the translational potential and limitations of each approach.

### 5.1 Controlled Drug Delivery Systems

Hydrogel drug delivery systems exploit the porous network structure and tunable degradation behavior to achieve controlled and targeted therapeutic delivery. The hydrophilic nature of hydrogels enables high water content and compatibility with diverse therapeutic payloads, including small molecules, proteins, nucleic acids, and nanoparticles.

#### 5.1.1 Small Molecule Delivery

Small‐molecule delivery from hydrogels relies primarily on diffusion‐controlled release, where the drug diffuses through the hydrated polymer network governed by Fickian diffusion principles. The release rate is determined by drug molecular weight, hydrogel mesh size, drug‐polymer interactions, and degradation behavior [135, 136]. Hydrophobic drugs require strategies including encapsulation in hydrophobic domains, formulation with surfactants or cyclodextrins, or covalent conjugation to the polymer network to achieve sustained release [137]. Ionizable drugs exhibit pH‐dependent release, enabling stimuli‐responsive delivery strategies.

Incorporation strategies for small molecules include physical encapsulation during gelation, postgelation loading through absorption from concentrated solutions, and covalent conjugation to polymer or crosslinker molecules. Each approach offers distinct advantages and limitations regarding loading efficiency, release kinetics, and drug activity preservation. Antibiotics, anti‐inflammatory agents, antioxidants, and pro‐regenerative molecules have all been successfully delivered from hydrogel systems.

#### 5.1.2 Growth Factor Delivery

Growth factor delivery presents significant challenges due to the labile nature, short half‐life, and complex bioactivity of these signaling molecules. Hydrogels protect growth factors from degradation and provide sustained, localized presentation that mimics physiological growth factor gradients [138]. Controlled release can be achieved through physical encapsulation, affinity‐based binding, or covalent conjugation.

Physical encapsulation of growth factors within hydrogel networks allows passive diffusion‐controlled release, though the release profile is often characterized by an initial burst followed by sustained release. The burst release can be mitigated through encapsulation in microspheres, complexation with heparin or other glycosaminoglycans, or covalent conjugation [139]. Affinity‐based delivery systems exploit specific interactions between growth factors and binding domains incorporated into the hydrogel. Heparin‐binding growth factors, including VEGF, FGF, and hepatocyte growth factor (HGF), can be retained through heparin conjugation to the polymer network, providing sustained release controlled by degradation or competitive displacement [138]. This approach more closely mimics the natural ECM sequestration of growth factors. Covalent conjugation of growth factors to hydrogel networks enables stable presentation of signaling molecules, with release dependent on network degradation or enzymatic cleavage of specific linker sequences. While covalent conjugation prevents burst release and ensures sustained presentation, it may reduce bioactivity through loss of mobility or altered conformation.

#### 5.1.3 Nucleic Acid Delivery

The delivery of nucleic acids, including DNA, siRNA, and mRNA, from hydrogels offers opportunities for gene‐based regenerative therapy [54]. Hydrogels protect nucleic acids from degradation, provide sustained release, and can be localized to specific tissue sites. Complexation of nucleic acids with cationic polymers, lipids, or inorganic nanoparticles enhances transfection efficiency and controls release kinetics.

Plasmid DNA delivery from hydrogels enables sustained protein expression for weeks to months, providing a depot of therapeutic gene expression [140]. siRNA delivery enables targeted gene silencing, with applications in modulating inflammation, inhibiting fibrosis, and controlling cell differentiation. mRNA delivery offers transient protein expression without genomic integration, reducing safety concerns while achieving therapeutic protein production [141].

The incorporation of nucleic acid carriers into hydrogels can enhance transfection efficiency while controlling release kinetics. Smart delivery systems that respond to cellular cues or enzymatic activity can achieve spatiotemporally controlled gene expression.

#### 5.1.4 Nanoparticle and Vesicle Delivery

Nanoparticle‐loaded hydrogels combine the advantages of controlled delivery with enhanced therapeutic loading and targeting capabilities. Polymeric nanoparticles, liposomes, solid lipid nanoparticles, and inorganic nanoparticles can be incorporated into hydrogel networks, providing additional control over release kinetics and enabling the delivery of payloads that would otherwise rapidly diffuse from the hydrogel [49]. The encapsulation of nanoparticles within hydrogels offers multiple advantages, including protection of sensitive payloads, controlled release through diffusion or matrix degradation, and the ability to incorporate multiple therapeutic agents with distinct release profiles [142]. Nanoparticles within hydrogels can also provide additional functionality, including photothermal therapy, magnetic targeting, or imaging contrast. Extracellular vesicles, including exosomes and microvesicles, have emerged as promising therapeutic vehicles due to their natural role in intercellular communication, reduced immunogenicity compared with cells, and ability to deliver complex payloads including proteins and nucleic acids [142]. Hydrogel delivery of extracellular vesicles provides sustained, localized presentation that enhances therapeutic efficacy in wound healing, cardiac repair, and neurological applications.

### 5.2 Cell Delivery and Encapsulation

Cell delivery from hydrogels represents a paradigm shift in regenerative medicine, moving from the delivery of soluble factors to the delivery of living therapeutic cells. Hydrogels protect delivered cells from shear stress, anoikis, and immune attack while providing a permissive microenvironment that supports cell survival, function, and integration.

#### 5.2.1 Mesenchymal Stem Cell Delivery

Mesenchymal stem cells (MSCs) are the most widely investigated cell type for hydrogel‐based delivery, reflecting their accessibility, immunomodulatory properties, and multipotent differentiation capacity [143]. MSC delivery from hydrogels has been investigated for applications spanning bone, cartilage, adipose, muscle, nerve, and cardiovascular tissues. The therapeutic efficacy of MSC delivery is increasingly attributed to paracrine mechanisms rather than direct differentiation, with MSCs secreting trophic factors that promote angiogenesis, modulate inflammation, and support endogenous tissue regeneration [144]. Hydrogel properties critically influence MSC behavior and therapeutic efficacy. Matrix stiffness modulates MSC differentiation, with soft matrices favoring neurogenic and adipogenic lineages, intermediate stiffness favoring myogenic lineages, and stiff matrices favoring osteogenic lineage [144]. Degradation kinetics influence MSC remodeling and tissue integration, with cell‐mediated degradation enabling space for newly deposited ECM. The incorporation of cell‐adhesive motifs, including RGD, facilitates MSC adhesion, spreading, and survival.

#### 5.2.2 Stem Cell Encapsulation Strategies

Cell encapsulation techniques must balance protection of cell viability with appropriate cell–material interactions. Bulk encapsulation, where cells are mixed with polymer solution before gelation, provides the most straightforward approach but offers limited control over cell distribution and may expose cells to toxic crosslinking conditions. Microencapsulation, where individual cells or small cell aggregates are encapsulated in hydrogel microspheres, provides improved mass transport, protection from immune attack, and injectability [145]. Core‐shell encapsulation approaches, where cells are encapsulated in a protective inner core surrounded by a selective outer membrane, provide immuno‐isolation while permitting nutrient and waste transport [146]. Multilayered encapsulation strategies provide additional control over the cellular microenvironment and immune protection.

#### 5.2.3 Alternative Cell Sources

Alternative cell sources for hydrogel‐based delivery include induced pluripotent stem cells (iPSCs), embryonic stem cells, tissue‐specific progenitor cells, and immune cells [147]. iPSCs offer patient‐specific therapy with unlimited expansion capacity, though their tumorigenic potential and differentiation variability remain challenges. Tissue‐specific progenitor cells, including osteoblasts, chondrocytes, and neural stem cells, provide lineage‐committed cells with established differentiation pathways [148]. Immune cell delivery, including regulatory T cells, dendritic cells, and macrophages, represents an emerging strategy for immunomodulation. Hydrogel delivery of immunomodulatory cells can enhance immune tolerance in transplant settings, reduce inflammation in autoimmune disease, and promote pro‐regenerative immune responses in tissue injury.

#### 5.2.4 Stem Cell‐Secretome Delivery

The recognition that stem cell therapeutic effects are largely mediated by paracrine factors has motivated the development of stem cell‐secretome delivery strategies that avoid direct cell‐DELIVERY challenges [149]. Conditioned media from stem cell cultures, containing growth factors, cytokines, chemokines, and extracellular vesicles, can be incorporated into hydrogels for therapeutic delivery. Secretome‐loaded hydrogels offer advantages including reduced safety concerns (no risk of tumorigenesis or immune rejection), simplified regulatory pathways (classified as biological products rather than cell therapies), and reduced manufacturing complexity [150]. The sustained release of the complex secretome repertoire may more accurately recapitulate the therapeutic effects of cellular therapies than delivery of individual factors.

### 5.3 Immunomodulatory Strategies

The immune response to implanted hydrogels critically influences therapeutic outcomes, with chronic inflammation leading to fibrosis and implant failure, while controlled immunomodulation can promote tissue integration and regeneration [10]. Hydrogels can be designed to actively modulate immune responses through passive and active strategies.

Passive immunomodulation exploits the intrinsic properties of hydrogel materials to minimize inflammatory responses. Chemically inert polymers, including PEG and PVA, resist protein adsorption and reduce inflammatory cell recruitment [9]. Hydrogel degradability can modulate the foreign body response, with biodegradable materials generally eliciting less severe chronic inflammation than non‐degradable materials. Surface charge, hydrophobicity, and topography influence protein adsorption, macrophage polarization, and lymphocyte activation. Active immunomodulation incorporates specific signals that promote pro‐regenerative immune responses. Incorporation of IL‐10, IL‐4, or IL‐13 can promote M2 macrophage polarization and anti‐inflammatory responses [151]. Delivery of T regulatory cell‐inducing signals, including transforming growth factor‐beta (TGF‐β) and retinoic acid, can promote immune tolerance. Hydrogels can also deliver immunosuppressive agents, including glucocorticoids and calcineurin inhibitors, to reduce inflammation in specific applications [152].

Macrophage polarization represents a key target for immunomodulatory hydrogels. Hydrogel properties, including stiffness, degradation products, and incorporated signals, influence whether macrophages adopt pro‐inflammatory M1 or pro‐regenerative M2 phenotypes [153]. Hydrogels that promote M2 polarization generally support tissue integration and regeneration, while those promoting M1 polarization may induce fibrosis. Strategies for promoting M2 polarization include IL‐4/IL‐13 delivery, glucocorticoid incorporation, and controlled degradation products that mimic endogenous immune signals.

### 5.4 Stimuli‐Responsive Behaviors

Stimuli‐responsive hydrogels exploit environmental triggers to achieve temporally and spatially controlled therapeutic delivery, network degradation, or mechanical property changes [154, 155]. While Section 3.2.4 describes the synthetic polymer materials that exhibit stimuli‐responsive properties, this section focuses on the functional strategies and therapeutic applications enabled by these behaviors. This intelligent behavior mimics aspects of the dynamic, responsive nature of native tissues and enables sophisticated therapeutic strategies.

pH‐responsive hydrogels incorporate ionizable groups, typically weak acids or bases, that undergo protonation or deprotonation at specific pH values, causing swelling or deswelling [122]. This behavior can be exploited to release therapeutic payloads in response to the acidic microenvironment of wounds or inflammation. pH‐responsive hydrogels have been developed for gastrointestinal drug delivery, where they protect payloads from gastric acid and release them in the higher pH of the intestine, and for wound healing applications, where they release antimicrobial agents in infected wounds characterized by acidic pH [156].

Temperature‐responsive hydrogels undergo sol–gel transitions at specific temperatures, enabling injectable formulations that gel at body temperature. PNIPAAm hydrogels, exhibiting a lower critical solution temperature around 32°C, enable low‐viscosity injection followed by rapid gelation at physiological temperature [157]. Thermoresponsive natural polymers, including methylcellulose and chitosan copolymers, offer alternative temperature‐responsive behavior with improved biocompatibility [158].

Enzyme‐responsive hydrogels incorporate substrates for cell‐secreted proteases, enabling cell‐mediated degradation and remodeling [159]. MMP‐sensitive hydrogels, incorporating peptide sequences recognized by matrix metalloproteinases, are degraded in response to cell activity, creating space for tissue infiltration and remodeling [86]. This cell‐responsive degradation couples scaffold persistence to cellular demand, potentially improving regenerative outcomes.

Photo‐responsive hydrogels undergo structural or functional changes upon light exposure, enabling precise spatiotemporal control over therapeutic delivery and network properties [160]. Photosensitive hydrogel systems can release therapeutic payloads, crosslink or degrade specific network components, or undergo reversible property changes. The use of near‐infrared light enables deeper tissue penetration, while visible and UV light enable precise spatial control in surface applications.

Energy‐responsive therapeutic hydrogels convert external energy inputs (light, ultrasound, or radiation) into localized therapeutic effects. Photothermal hydrogels incorporate nanomaterials such as polydopamine, MXene, or gold nanoparticles that generate localized heat under near‐infrared irradiation, enabling antibacterial therapy and controlled drug release from thermosensitive components [161]. Photodynamic hydrogels encapsulate photosensitizers such as chlorin e6 or methylene blue that generate reactive oxygen species upon light activation for antimicrobial and biofilm eradication applications [162]. Sonodynamic hydrogels deliver sonosensitizers activated by ultrasound, enabling therapeutic effects in deep tissues where light penetration is insufficient [163]. Radiotherapy‐enhancing hydrogels incorporate radiosensitizers or oxygen‐generating nanoparticles to improve radiation sensitivity in tissue contexts where adjuvant therapy is required [164]. These energy‐responsive systems enable localized, spatiotemporally controlled therapy that minimizes systemic toxicity.

Dual and multiresponsive hydrogels combine multiple stimuli‐responsive behaviors, enabling sophisticated therapeutic strategies tailored to specific physiological contexts. Dual pH/temperature‐responsive hydrogels can deliver therapeutics in response to both the acidic environment of wounds and body temperature [165]. Multiresponsive systems can achieve sequential therapeutic delivery corresponding to different phases of wound healing or disease progression [166].

### 5.5 Critical Assessment of Bioactive Integration Strategies

Although bioactive integration has significantly expanded the therapeutic potential of multifunctional hydrogels, clinical translation remains limited. Many strategies that demonstrate promising outcomes in preclinical models face challenges related to dose control, biological complexity, manufacturing, and reproducibility. Therefore, a critical evaluation of current approaches is essential to identify strategies with genuine translational potential.

Growth factor delivery represents one of the most extensively investigated approaches, yet its clinical success remains modest. Despite decades of research, only limited growth factor‐based products have reached clinical use, highlighting the complexity of translating these molecules into effective therapies. Their therapeutic activity depends strongly on dosage, timing, localization, and interactions with the surrounding microenvironment. Conventional hydrogel delivery systems often struggle to achieve precise and sustained presentation due to burst release, insufficient loading efficiency, and enzymatic degradation. Moreover, prolonged exposure to certain growth factors may produce undesirable effects depending on the tissue context [138]. These limitations have encouraged the development of affinity‐based delivery systems that mimic ECM‐mediated sequestration, as well as alternative strategies based on stem cell secretomes and extracellular vesicles that provide more complex and physiologically relevant signaling profiles.

Cell‐based hydrogel therapies face additional translational barriers. Although hydrogels can improve cell retention and provide protective niches, transplanted cells frequently exhibit limited survival due to oxidative stress, immune responses, and insufficient vascular support. Furthermore, cell manufacturing, quality control, immunogenicity, and regulatory requirements significantly complicate clinical application. Increasing evidence suggests that the therapeutic effects of many stem cell therapies are primarily mediated by paracrine factors rather than direct tissue replacement [144]. Furthermore, due to the complexity of the evaluation and approval process and for cell therapy, cell‐free approaches, including extracellular vesicle and secretome delivery, have emerged as promising alternatives [148, 150]. However, challenges related to isolation standardization, characterization, potency evaluation, and mechanism clarification remain unresolved.

Immunomodulatory strategies have also become central to multifunctional hydrogel design, but current approaches require more precise biological understanding. The traditional M1/M2 macrophage polarization model provides a simplified framework but does not fully reflect the dynamic and heterogeneous nature of immune responses in vivo. Promoting a regenerative macrophage phenotype is not universally beneficial, as immune functions vary depending on tissue type, disease stage, and regeneration requirements [151, 153]. Future immunomodulatory hydrogels should therefore focus on regulating specific dysfunctional pathways, such as inflammasome activation, efferocytosis, or immune–matrix interactions, rather than simply inducing broad macrophage polarization. Integration of immune regulation with antioxidant protection, angiogenesis, and matrix remodeling may provide more effective therapeutic outcomes.

The combination of multiple therapeutic functions within a single hydrogel platform represents both the major advantage and challenge of multifunctional systems. Integrating growth factors, cells, nanoparticles, and responsive elements can address the multifactorial nature of tissue injury; however, increased complexity also complicates mechanistic interpretation, manufacturing scalability, quality control, and regulatory evaluation. Therefore, multifunctionality should not be pursued through the accumulation of independent components but through rational integration of complementary functions with clear therapeutic benefits. Future successful platforms will likely be those achieving an optimal balance between biological sophistication, manufacturing feasibility, and clinical practicality rather than simply maximizing material complexity.

## 6 Therapeutic Applications Across Regenerative Contexts

The rational design principles and bioactive integration strategies discussed in the preceding sections find their ultimate expression in the diverse therapeutic applications of multifunctional hydrogels across tissue types. This section provides a comprehensive overview of hydrogel‐based regenerative strategies for wound healing and skin regeneration, bone and cartilage repair, neural regeneration, cardiovascular tissue engineering, and emerging multitissue applications, highlighting the specific design requirements, therapeutic cargoes, and preclinical outcomes for each context.

### 6.1 Wound Healing and Skin Regeneration

Cutaneous wound healing represents one of the most clinically advanced applications of multifunctional hydrogels, reflecting the accessible wound site, well‐defined healing phases, and significant unmet clinical need [167]. Hydrogels address multiple aspects of wound healing, including moisture management, infection control, inflammation modulation, and tissue regeneration. Acute wound management benefits from hydrogel properties including moisture retention, exudate absorption, and infection protection [168]. Simple hydrogels, including alginate, gelatin, and synthetic polymers, provide a moist wound environment that supports re‐epithelialization and reduces pain. Antibiotic‐releasing hydrogels address infection risk, while antioxidant hydrogels can modulate oxidative stress. Photo‐crosslinkable hydrogels enable in situ gelation, conforming to irregular wound geometries [168]. Chronic wounds, including diabetic ulcers, venous ulcers, and pressure ulcers, demand more sophisticated therapeutic strategies [169]. Chronic wounds are characterized by persistent inflammation, impaired angiogenesis, and elevated protease activity, necessitating multifunctional hydrogels that address multiple pathological features. Consequently, successful hydrogel platforms increasingly integrate antimicrobial activity, antioxidant protection, immune regulation, and angiogenic stimulation rather than relying on single therapeutic mechanisms [96].

Growth factor delivery has been extensively investigated for chronic wound healing. Platelet‐derived growth factor (PDGF), epidermal growth factor (EGF), vascular endothelial growth factor (VEGF), and fibroblast growth factor (FGF) have all been delivered from hydrogels, with varying degrees of success [170]. However, the complex pathophysiology of chronic wounds often requires multifactor approaches. Stem cell and stem cell‐secretome delivery from hydrogels offers more comprehensive therapeutic effects, addressing multiple aspects of wound pathology through paracrine mechanisms [96]. Immunomodulatory hydrogels that promote M2 macrophage polarization and resolve chronic inflammation represent emerging strategies for chronic wound healing. Advanced wound dressing formats include sheet hydrogels, sprayable formulations, and in situ gelling systems [171]. The integration of hydrogels with wearable sensors and drug delivery systems enables closed‐loop wound management, where the dressing monitors wound status and adjusts therapeutic delivery accordingly [172]. Bioelectronic hydrogels that incorporate electrical stimulation may further enhance wound healing through mechanisms including increased cell migration and alignment [173].

Recent advances demonstrate diverse strategies for integrating multiple therapeutic functions within wound‐healing hydrogels. A representative NIR‐responsive system, the GelMA/CeO_2_/polydopamine hydrogel, integrates antioxidant cerium oxide nanoparticles with photothermal polydopamine to scavenge reactive oxygen species and provide controlled antibacterial activity under NIR stimulation (Figure 4D) [174]. In diabetic rat models, this dressing significantly accelerated wound closure by modulating the IL‐17 signaling pathway while effectively reducing bacterial burden against both *E. coli* and MRSA. Similarly, Sanghuang polysaccharide and MXene nanosheet hydrogels within dopamine‐modified chondroitin sulfate and GelMA‐PBA networks have achieved photoresponsive antibacterial activity, ROS scavenging, and M2 macrophage polarization for infected wound healing [175]. Bacterial cellulose‐polyvinyl alcohol‐borax/GelMA hydrogels functionalized with tannic acid‐modified MXene nanosheets further demonstrated self‐healing properties and photothermal antibacterial activity under NIR irradiation [176]. Despite these promising results, reliance on external light sources limits clinical practicality for deep or irregular wounds, and the long‐term biosafety of photothermal agents such as MXene remains inadequately characterized.

**FIGURE 4:**
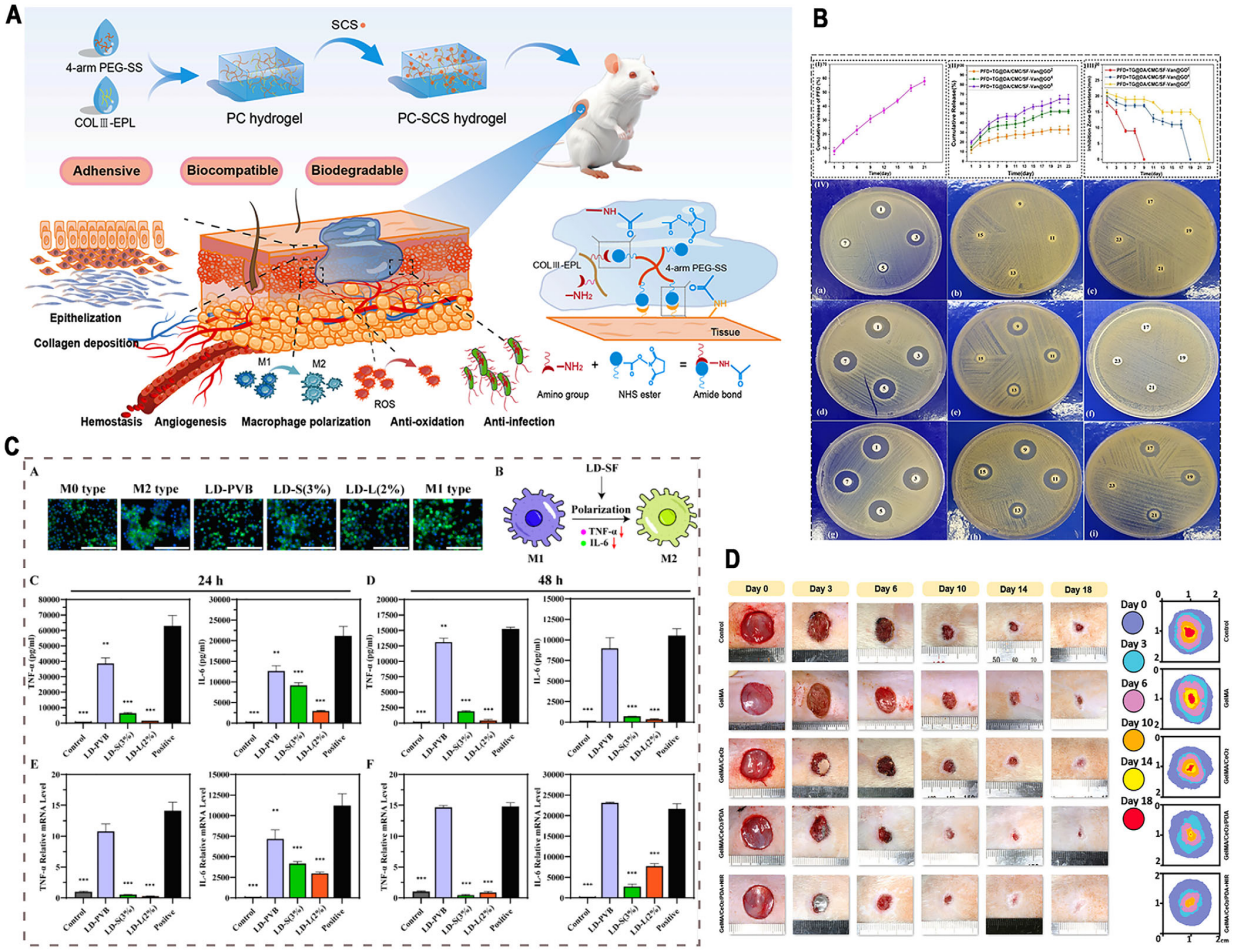
Multifunctional hydrogels application in Wound healing. (A) Schematic illustration of PC‐SCS hydrogel promoting diabetic wound healing. Reproduced with permission [177]. Copyright 2025, Elsevier. (B) Drug delivery and antimicrobial ability of PFD + TG@DA/CMC/SF hydrogels with different concentrations of Van@GO. Reproduced with permission [178]. Copyright 2025, Elsevier. (C) In vitro immunomodulatory evaluation of silk fibroin hydrogel dressing (LD‐SF): cytoskeleton staining of RAW 264.7 macrophages (blue: DAPI, green: FITC‐phalloidin), schematic of M1‐to‐M2 macrophage polarization, and TNF‐α/IL‐6 expression at protein and gene levels after LPS stimulation (mean ± SEM, *n* = 3). Reproduced with permission [179]. Copyright 2025, Elsevier. (D) Representative images of full‐thickness skin defects in diabetic rats treated with GelMA/CeO_2_/PDA hydrogel on days 0, 3, 6, 10, 14, and 18, with superimposed wound outlines for all groups (colors indicate days). Reproduced with permission [174]. Copyright 2025, Wiley‐VCH.

An alternative paradigm emphasizes sustained delivery of bioactive therapeutics to regulate the wound microenvironment. A magnesium‐doped bioactive glass hydrogel programs mesenchymal stem cell‐derived small extracellular vesicles into vesicle clusters (EPPM), enhancing intracellular sEV uptake by 8.2‐fold in macrophages and 16.7‐fold in endothelial cells, promoting M2 polarization, reducing inflammation, and accelerating angiogenesis and collagen deposition in diabetic wound models [180]. Similarly, glucose‐responsive PEGDA‐GelMA‐MPBA hydrogels achieve on‐demand exosome release under hyperglycemic conditions through dynamic boronate ester bonds, reducing inflammation and enhancing VEGF‐mediated angiogenesis [181]. Sustained co‐delivery of Panax notoginseng saponins and IGF‐1 from GelMA/PNS/Alg@IGF‐1 hydrogels further reduces oxidative stress, suppresses senescence markers, inhibits NF‐κB activation, and restores endothelial function under diabetic conditions [182]. Despite these encouraging outcomes, challenges persist regarding extracellular vesicle standardization, optimal dosing regimens, and whether vesicle‐based approaches justify their complexity relative to purified growth factors.

Structural engineering has further expanded hydrogel functionality by integrating bioactive nanomaterials and ECM‐inspired components. Injectable silk fibroin methacryloyl hydrogels encapsulating pH‐responsive zinc sulfide nanoparticles release therapeutic H_2_S and Zn^2^^+^ ions under acidic wound conditions, demonstrating potent ROS‐scavenging capacity, promoting M2 macrophage polarization, and accelerating wound closure, re‐epithelialization, and neovascularization in diabetic models [183]. Dopamine‐functionalized hyaluronic acid and recombinant human type III collagen hydrogels crosslinked via an H_2_O_2_/HRP catalytic system (HA‐DA@rhCol) combine tissue adhesion, antioxidant activity, photothermal antibacterial effects, and angiogenic stimulation for vascularized wound repair [184]. Collagen‐fucoidan hydrogels reinforced with glutaraldehyde‐derived carbon dots down‐regulate TNF‐α and IL‐1β while promoting TGF‐β1 expression, working synergistically for antibacterial and anti‐inflammatory effects [185]. Injectable collagen‐silver nanoparticle hydrogels fabricated through a one‐step visible light‐induced strategy eliminate toxic reducing agents while providing broad‐spectrum antimicrobial activity and bioactive regenerative scaffold function [186]. Cellulose nanofiber/polyvinyl alcohol hydrogels incorporating curcumin‐modified silver nanoparticles exhibit high mechanical strength (tensile 231 kPa, compressive 1.23 MPa), self‐healing, tissue adhesion, and >98% cell viability for chronic wound repair [187]. While these platforms demonstrate that mechanical reinforcement and biological functionality can be simultaneously achieved, they introduce formulation complexity that complicates manufacturing reproducibility and regulatory evaluation.

Addressing the mechanical demands of dynamic wounds, injectable and adhesive systems have been designed to provide secure wound coverage under challenging conditions. Dopamine‐grafted tragacanth/carboxymethyl chitosan/silk fibroin hydrogels containing vancomycin‐functionalized graphene oxide and pirfenidone combine sustained antibacterial delivery, antioxidant activity, electrical conductivity, and antifibrotic effects, enhancing angiogenesis while minimizing scar formation in MRSA‐infected burn wounds (Figure 4B) [178]. An in situ‐forming silk fibroin/polyvinyl butyral hydrogel demonstrates molecular‐weight‐dependent bioactivity: low‐molecular‐weight SF (45 kDa) promotes fibroblast proliferation, while high‐molecular‐weight SF (72 kDa) polarizes macrophages toward pro‐resolving phenotypes, orchestrating immunomodulation and ECM regeneration with minimized scarring (Figure 4C) [179]. A bioengineered Tetra‐PEG‐COLIII‐SCS hydrogel integrates wet adhesion, rapid hemostasis, antioxidant activity, and HIF‐1α/VEGF‐mediated angiogenesis, promoting scarless diabetic wound healing through regulated collagen I/III deposition (Figure 4A) [177].

These studies demonstrate that effective wound regeneration increasingly depends on the rational integration of complementary therapeutic functions rather than single‐mechanism interventions.

### 6.2 Bone and Cartilage Regeneration

The unique mechanical demands of musculoskeletal tissues require hydrogels with tailored mechanical properties, degradation kinetics, and biological cues [188]. Bone regeneration demands coordination between mechanical support, osteogenic signaling, vascularization, and immune regulation, prompting multifunctional hydrogels that combine osteoinductive cues with angiogenic and immunomodulatory functions. Bone morphogenetic proteins, particularly BMP‐2, have been extensively investigated with strategies for sustained, localized delivery that minimizes off‐target effects [189], while hydroxyapatite and calcium phosphate nanoparticle incorporation provide osteoconductivity alongside mechanical reinforcement. Cartilage regeneration presents distinct challenges due to its avascular nature and limited self‐repair capacity [190]. Hydrogel strategies typically involve chondrocyte or mesenchymal stem cell delivery, with mechanical properties matching native cartilage compressive and shear properties to support load‐bearing while promoting chondrogenesis. Injectable hydrogels that gel in situ and fill irregular defect geometries are particularly attractive for articular cartilage repair [191]. Osteochondral regeneration demands spatially resolved formulations addressing the distinct requirements of both tissues [192], with gradient or bilayered hydrogels providing discrete bone and cartilage compartments optimized for respective regeneration. Intervertebral disc degeneration represents another target for injectable therapies delivering cells or factors to restore disc height and function, with the nutrient‐poor environment demanding controlled degradation and sustained release [83].

Recent advances in osteochondral interface engineering have focused on replicating the hierarchical structure of native tissues. A nanoscale biomimetic bilayer hydrogel incorporating mineralized and nonmineralized collagen fibrils embedded with tendon stem cells promoted fibrocartilage formation, improved motor function, and enhanced biomechanical outcomes in patella‐patellar tendon‐interface injury models [193]. Similarly, bioinspired in situ‐forming hyaluronic acid‐chondroitin sulfate‐collagen short nanofiber hydrogels achieved tunable gelation time (11–360 s) and compressive modulus (1.4–16.2 kPa) while supporting chondrogenic differentiation without severe inflammation [194].

Exosome‐based strategies have emerged for cartilage repair. An injectable photocrosslinkable hydrogel composed of o‐nitrobenzyl alcohol‐modified hyaluronic acid and gelatin loaded with shRNA‐EZH2‐modified exosomes (ExoshEZH2@HG) exhibited rapid gelation, cancellous bone‐like compressive strength, and controlled exosome release, inhibiting EZH2 overexpression and NF‐κB signaling while promoting BMSC migration and chondrogenesis, ultimately facilitating hyaline cartilage‐like regeneration in rat defect models [195]. A ginsenoside CK‐hybridized exosome composite macroporous hydrogel scaffold (HyExo@CK/SiCH) featuring interconnected macropores (171.40 ± 7.37 µm) coordinated endogenous BMSC recruitment, retention, and postdifferentiation fate regulation by competitively binding to the SDF‐1 ligand‐binding domain, achieving complete cartilage regeneration within 12 weeks in rabbit osteoarthritis models (Figure 5A) [196]. Thermosensitive hydroxybutyl chitosan hydrogels co‐loaded with strontium acetate and chondroprogenitor‐derived exosomes (HBC@Sr@CPC‐EXOs) achieved time‐staggered release, with strontium mediating early anti‐inflammatory effects (0–12 h) and exosomes sustaining late‐phase immunoprogramming (24–48 h), activating TGF‐β1/Smad2/3 while suppressing Notch signaling to drive BMSC chondrogenesis and achieve superior osteochondral repair with higher ICRS scores [197]. A thermoresponsive hydroxybutyl‐chitosan hydrogel integrating iridium nanozymes, ZIF‐8, and kartogenin (HBC@ZIrK) preserved subchondral bone, reduced osteophytes, suppressed inflammatory cytokines, and enhanced cartilage matrix synthesis in TMJ‐OA models, achieving joint morphology statistically indistinguishable from healthy controls [198].

**FIGURE 5:**
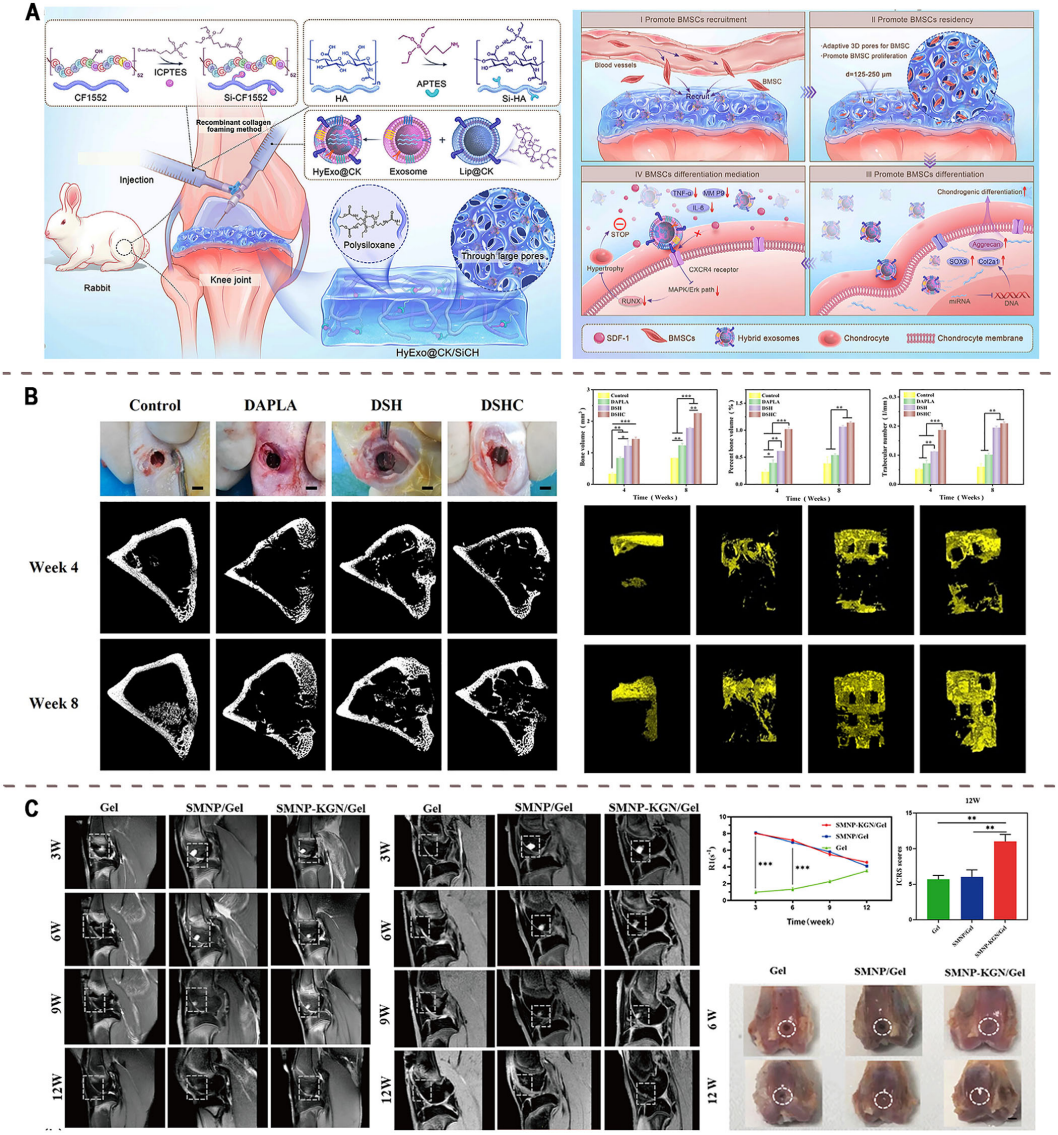
Multifunctional hydrogels application in Bone and Cartilage regeneration. (A) Design and Therapeutic efficacy of the HyExo@SiCH hydrogel in promoting osteoarthritis cartilage repair, illustrating BMSCs recruitment, retention, and differentiation regulation. Reproduced with permission [196]. Copyright 2026, Wiley‐VCH. (B) Rabbit tibial defect model establishment and micro‐CT‐based healing assessment (White: 2D micro‐CT images. Yellow: representative 3D reconstruction images from micro‐CT) of carboxymethyl chitosan‐enhanced multilevel microstructured composite hydrogel 3D scaffolds. Reproduced with permission [199]. Copyright 2025, Elsevier. (C) MRI and macroscopic analysis of cartilage regeneration by SMNP‐KGN/Gel hydrogel scaffold: MR proton‐density‐weighted imaging (PDWI) at 3–12 weeks, water‐selective cartilage scan (3D_WATSc) for hydrogel degradation monitoring, semiquantitative R_1_ relaxometry comparison, harvested cartilage observations at weeks 6 and 12, and ICRS scores for PBS, SMNP/Gel, and SMNP‐KGN/Gel groups. Reproduced with permission [200]. Copyright 2023, Wiley‐VCH.

Engineered hydrogels with enhanced mechanical properties have been developed for load‐bearing applications. An enzymatically cross‐linked gelatin‐oxidized starch nanocomposite hydrogel reinforced with lithium silicate nanoplatelets (GelS‐TG‐SN) achieved ultrafast gelation (<1 min), robust underwater adhesion, and significantly enhanced mechanical strength, supporting dual‐lineage differentiation of synovial‐derived stem cells, chondrogenesis in cartilage‐like niches and PI3K‐Akt‐mediated osteogenesis in vascular‐like niches, for coordinated osteochondral regeneration under arthroscopic conditions [201]. An injectable self‐healing alginate‐gelatin‐chondroitin sulfate hydrogel (OSAGC) with compressive strength up to 3 MPa and sustained release over 3 months promoted new bone formation within four weeks and new cartilage within twelve weeks [202]. Polyvinyl alcohol‐chitosan‐sodium alginate hydrogels achieved exceptional compressive strength (141 MPa), 93% creep recovery, and low friction coefficient (0.044), positioning them as ideal articular cartilage substitutes [203]. Citrate‐treated PVA/PAM double‐network hydrogels achieved tensile strength of 18.9 ± 1.6 MPa, compressive strength of 102.3 ± 7.9 MPa, and 99.2% stress retention after 500 fatigue cycles through Hofmeister effect‐induced densification, supporting chondrocyte adhesion and proliferation [204]. A hydroxyapatite cross‐linked in situ polyvinyl alcohol hydrogel achieved tensile strength of 1890 kPa, compressive strength of 1125 kPa, and durability through 100 compression‐recovery cycles for calcified cartilage layer repair [205].

Cartilage‐specific platforms have utilized silk fibroin and interpenetrating network strategies. A methacrylate‐modified silk fibroin hydrogel embedded with kartogenin exhibited porous architecture and chondrogenic differentiation‐promoting activity, demonstrating remarkable superficial cartilage repair capacity [206]. Gelatin methacrylate‐silk fibroin interpenetrating network hydrogels achieved enhanced mechanical strength (compressive modulus 300 kPa), reduced swelling, and supported BMSC chondrogenesis with elevated Col‐2, Acan, and Sox‐9 expression, with cell‐seeded hydrogels promoting satisfactory cartilage repair in vivo [207]. An RGD‐modified hyaluronic acid methacryloyl hydrogel co‐loaded with kartogenin and Klotho (HRKK) attenuated oxidative stress, promoted anti‐inflammatory macrophage polarization, and recruited BMSCs while preserving chondrogenesis under inflammation, establishing a regenerative niche through sequential Klotho and KGN delivery [208].

Bone regeneration platforms have integrated 3D printing and biomimetic ECM strategies. A multilevel microstructured composite scaffold integrating 3D‐printed PLA with sodium alginate/nano‐hydroxyapatite/carboxymethyl chitosan hydrogel increased bone trabecular number 2.06‐fold in rabbit tibial defects, with CMCS enhancing crosslinking, mineralization, and osteogenic activity (Figure 5B) [199]. A cellulose nanofiber‐reinforced alginate‐gelatin hydrogel achieved injectability (∼150 s gelation), high compressive modulus (361.3 kPa), and 92% self‐healing efficiency with excellent biomineralization (Ca/P ∼1.69) and >96% preosteoblast viability [209]. An ECM‐mimicking GelMA‐DNA interpenetrating network hydrogel with aptamer‐mediated BMSC recruitment and sustained VEGF release promoted osteogenic differentiation through FAK/PI3K/Akt/β‐Catenin activation, enhancing vascularized bone regeneration [210]. A carbon nanotube‐doped peptide hydrogel‐PCL composite scaffold with tunable mechanical properties and conductivity promoted osteogenesis and chondrogenesis through increased mineralization, ALP activity, and GAG secretion, facilitating accelerated cartilage and subchondral bone regeneration in rabbit knee defects [211]. Theranostic cellulose nanocrystal/GelMA/HAMA hydrogels loaded with synthetic melanin nanoparticles and kartogenin enabled MRI‐guided cartilage regeneration with sustained KGN release promoting BMSC chondrogenesis while allowing simultaneous monitoring over 12 weeks (Figure 5C) [200].

Collectively, these studies demonstrate that effective musculoskeletal regeneration increasingly depends on integrating mechanical reinforcement, biochemical signaling, and immunomodulation within single platforms [212].

### 6.3 Neural Regeneration

Neural tissue regeneration presents extraordinary challenges, including the limited regenerative capacity of the adult central nervous system, the need for precise axonal guidance, and the complexity of the neural microenvironment [213]. Successful regeneration requires structural guidance, electrical, biochemical, and immunological regulation, making multifunctional hydrogels attractive platforms for integrating conductive elements, neurotrophic factors, and anti‐inflammatory strategies. Spinal cord injury hydrogels must bridge the injury site, provide a permissive substrate for axonal growth, and modulate the inhibitory glial scar [214]. Neurotrophic factors including nerve growth factor (NGF), brain‐derived neurotrophic factor (BDNF), glial cell line‐derived neurotrophic factor (GDNF), and neurotrophin‐3 (NT‐3) have been delivered from hydrogels with gradient release strategies guiding axonal growth toward appropriate targets. Peripheral nerve regeneration hydrogels typically take the form of nerve guidance conduits providing a physical scaffold across a gap [215], with Schwann cells, stem cells, or neurotrophic factors enhancing regeneration while controlled degradation ensures persistence. Traumatic brain injury and stroke represent additional applications, with injectable hydrogels filling cavitary defects, delivering neuroprotective agents, and supporting endogenous neurogenesis [216, 217], while immunomodulatory hydrogels reducing neuroinflammation are particularly relevant for stroke, where secondary inflammatory damage exacerbates injury.

Guidance of neural stem cell migration represents a foundational strategy for neural tissue engineering. A gradient hydrogel scaffold incorporating glutamic acid‐modified hyaluronic acid, gelatin methacryloyl, sodium carboxymethyl cellulose, and collagen was fabricated through light‐curing printing with an interpenetrating polymer network featuring vascular‐like fibrillar collagen and brain‐mimetic lamellar HA‐Glu structures. The system achieved rapid photo‐crosslinking within 75 seconds with cell viability exceeding 85%, and gradient hydrogels with varying HA‐Glu/collagen concentration regions directionally guided cellular migration toward regions with higher component concentrations, providing practical insights for multilayer neural tissue engineering [218]. Similarly, an on‐demand injectable hyaluronic acid‐DNA hydrogel engineered through a dual‐tube hybrid‐bridge design with sequence‐level randomization (*N* = 8 randomized bases) prevented premature network formation while enabling rapid homogeneous gelation upon precursor mixing, supporting uniform neural progenitor cell encapsulation with high viability and sustained SOX2/NESTIN expression. The system exhibited shear‐thinning, rapid self‐healing, and robust postextrusion integrity for minimally invasive neural tissue engineering [219]. An alginate hydrogel‐based platform achieved high‐efficiency astrocytic differentiation from human neural stem cells within three days, drastically outperforming conventional methods requiring weeks. Mechanistically, spontaneous decrosslinking altered mechanical properties, activating Piezo1 Ca^2^^+^ channels and facilitating YAP nuclear translocation via the actomyosin cytoskeleton, promoting YAP/TEAD‐mediated transcription essential for astrocytic differentiation [220]. These studies demonstrate that both biochemical gradients and mechanical cues direct neural cell fate, though their relative contributions remain insufficiently characterized.

Immunomodulation has emerged as a central strategy for neural repair. A gelatin methacrylate hydrogel incorporating MgFe‐layered double hydroxide nanoparticles functionalized with BDNF enabled sustained neurotrophic release while LDH nanoparticles promoted neural stem cell differentiation, suppressed M1 microglia markers, and enhanced M2 polarization, significantly improving motor function in rat SCI models (Figure 6B) [221]. An injectable quaternized carboxymethyl chitosan‐hyaluronic acid hydrogel loaded with microglia‐derived exosomes overexpressing miR‐124‐3p promoted microglial M2 polarization through mTOR inhibition, improving locomotor coordination and memory in mouse controlled cortical impact models [222]. A multifunctional ECM hydrogel incorporating SDF‐1α‐loaded hyaluronic acid and decellularized brain ECM mimicked brain composition with injectable, self‐healing properties and brain‐matching mechanics, protecting immune cells from dysfunction, while SDF‐1α and brain matrix synergistically recruited endogenous neural stem/progenitor cells and induced neuronal differentiation, restoring limb balance, locomotion, spatial learning, memory, and mood in severe TBI models [223]. While these approaches demonstrate promise, achieving sustained immunoregulation without compromising beneficial inflammatory responses remains challenging, and whether exosome‐based or ECM‐mimetic approaches offer distinct advantages has not been systematically established.

**FIGURE 6:**
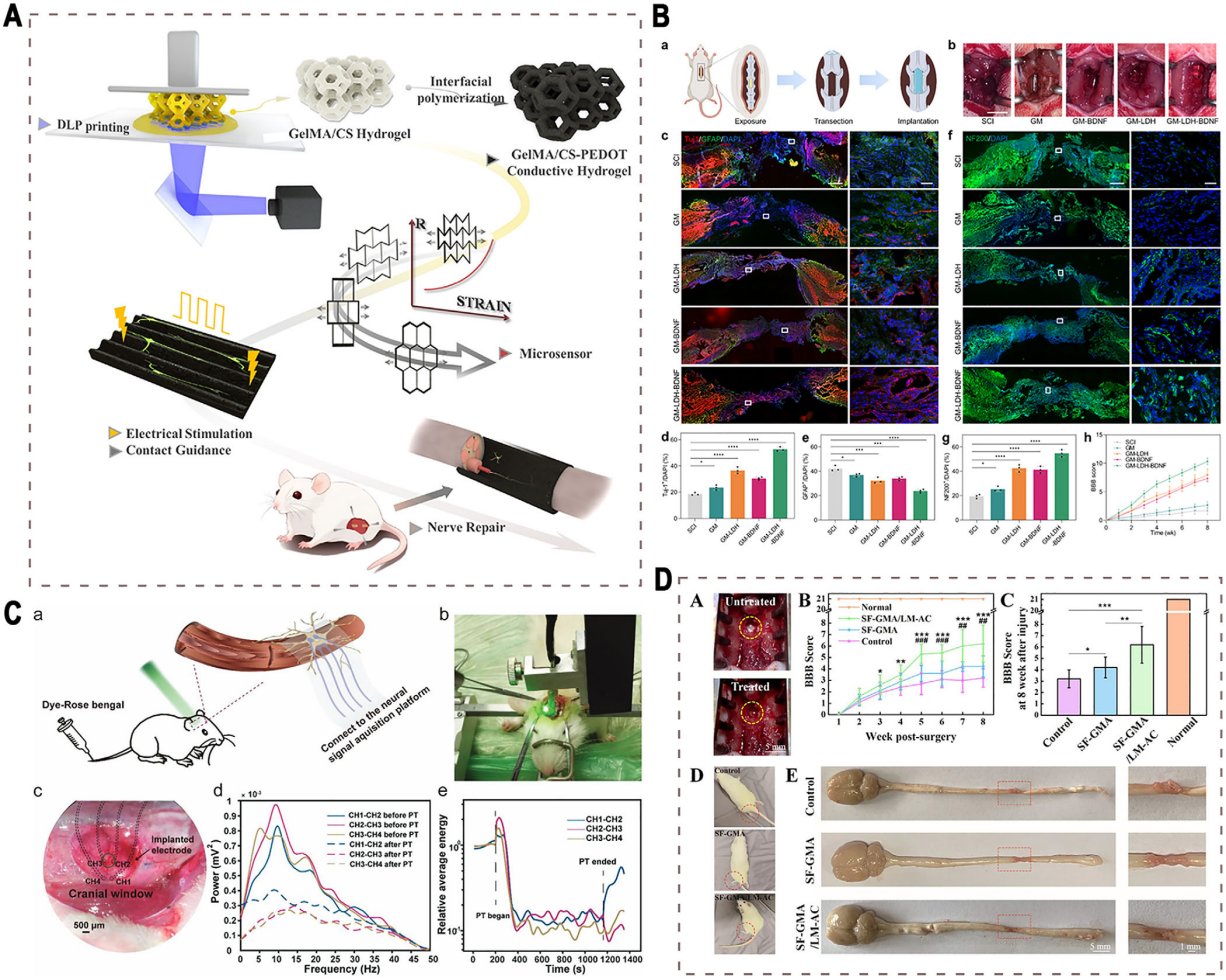
Multifunctional hydrogels application in Neural regeneration. (A) Schematic illustration of the fabrication process for conductive GelMA/CS‐PEDOT hydrogel achieved using DLP 3D printing for peripheral nerve injury repair. Reproduced with permission [224]. Copyright 2024, Elsevier. (B) In vivo evaluation of the reparative effects of MgFe‐LDH‐doped GelMA hydrogel scaffold in spinal cord injury. Reproduced with permission [221]. Copyright 2026, Elsevier. (C) Electrode‐based PEGylated Silk Fibroin Hydrogel (PSF) in situ characterization of neural activity in a rat photothrombosis acute stroke model. Reproduced with permission [225]. Copyright 2021, Wiley‐VCH. (D) The spinal cord anatomy and locomotor recovery of SCI rats for adhesive and SF‐GMA hydrogel. Reproduced with permission [20]. Copyright 2023, Elsevier.

Conductive hydrogels support both peripheral nerve regeneration and neural interfacing. A 3D‐printable gelatin methacryloyl/chitosan hydrogel incorporating conductive PEDOT nanoparticles via digital light processing enhanced electrical conductivity and mechanical properties, promoting PC12 and Schwann cell proliferation and axon outgrowth, with conductive nerve conduits repairing 10 mm rat sciatic nerve defects (Figure 6A) [224]. A transparent, stretchable hydrogel electrode based on PEGylated silk fibroin and PEDOT:PSS achieved ≈260% stretchability, ≈4‐month stable electrical performance, and low sheet resistance (≈160 Ω sq^−^^1^), enabling seamless brain contact and unobstructed optical interrogation with simultaneous rat‐brain imaging (Figure 6C) [225]. A modular biosynthetic PVA‐gelatin hydrogel crosslinked via visible light‐triggered thiol‐ene chemistry promoted astrocytic growth with brain‐compatible stability; co‐encapsulation with neural progenitors promoted neuronal differentiation and synaptically competent network formation, with constructs functioning as biohybrid interfaces *ex vivo* [226]. The long‐term stability of conductive components and potential for chronic inflammation remain critical translational concerns.

Adhesive and injectable systems provide stable interfaces with neural tissues. An in situ‐forming glycidyl methacrylated silk fibroin/lamininacrylate hydrogel formed a network rapidly upon UV triggering, with topological entanglement stitching the hydrogel to wet spinal cord tissue. Covalent lamininacrylate incorporation provided a superior microenvironment for neural stem cell growth, guiding neural regeneration over 8 weeks with hind limb locomotion recovery in rat complete transection SCI models (Figure 6D) [20]. A thermosensitive injectable fibrillar cellulose nanocrystal‐grafted poly(N‐isopropylacrylamide) hydrogel formed a fibrillar structure with brain‐mimetic mechanical properties (strain‐stiffening, compression softening), demonstrating biocompatibility with primary brain cells and enabling programmable paclitaxel release for postoperative glioblastoma treatment [227]. The long‐term biocompatibility of UV‐crosslinked systems and degradation product toxicity require further investigation.

These studies demonstrate that neural regeneration depends on integrating guidance cues, immunomodulation, electrical conductivity, and adhesive properties within hydrogel platforms.

### 6.4 Cardiovascular Tissue Engineering

Cardiovascular tissue engineering demands hydrogels with dynamic mechanical properties that match the cyclic loading of cardiac and vascular tissues [228]. Myocardial infarction (MI) hydrogels aim to limit infarct expansion, deliver cardioprotective and pro‐angiogenic factors, and support cardiac regeneration. Injectable hydrogels that gel in situ and conform to ventricular geometry can provide mechanical support that prevents adverse remodeling while delivering therapeutic payloads [229]. Stem cell delivery to the heart has been extensively investigated, with hydrogels improving cell retention and survival compared with direct injection [228], while growth factor delivery, including VEGF, HGF, and IGF‐1, promotes angiogenesis and cardiomyocyte survival [230]. Vascular graft hydrogels must support endothelial cell adhesion and function while resisting thrombosis and neointimal hyperplasia, with compliance matching native vessels to avoid hemodynamic complications [231, 232].

Phase‐specific therapeutic delivery has emerged as a strategy to address the sequential pathological events following myocardial infarction. A smart stimulus‐responsive dual‐crosslinked hydrogel enabled precise release across the three pathological phases of MI (inflammation, proliferation, and remodeling), rapidly releasing curcumin for anti‐apoptotic/anti‐inflammatory effects, rhCol III during proliferation for neovascularization, and RepSox (TGF‐β inhibitor) from PF127‐CHO@RepSox nanoparticles during remodeling to inhibit fibrosis, improving cardiac function and alleviating remodeling [233]. A paintable dextran‐aldehyde/gelatin hydrogel patch achieved in situ gelation within 135 s with cardiac tissue‐like modulus (40.5 kPa) and adhesiveness (4.3 kPa), enabling sustained ANGPTL4 release that increased vascularization, reduced inflammatory macrophages, and promoted cardiac cell maturation in acute MI models [234]. An injectable carboxymethyl chitosan‐dextran sulfate hydrogel with dynamic crosslinking mimicked myocardial ECM mechanics while integrating antioxidant, anti‐inflammatory, pro‐angiogenic, and conductive functions, scavenging ROS, protecting cardiomyocytes, modulating macrophage polarization, and promoting vascular regeneration with significant cardiac function improvement in rat MI models [235]. A MitoQ‐loaded gamma‐PGA/PEG hydrogel, evaluated as both patch and intramyocardial injection, showed slow MitoQ release reducing ROS and mitochondrial damage while enhancing cardiomyocyte activity, with intramyocardial injections demonstrating superior efficacy in cardiac function and structural restoration [236].

Mechanical matching of hydrogel properties to native myocardial tissue has proven critical for therapeutic efficacy. Three acellular hydrogel patches with tailored elastic moduli; soft (1.61 kPa), mechano‐matching (16.82 kPa, matching native myocardium), and rigid (602.61 kPa), revealed that the mechano‐matching patch improved cardiac function (LVEF +15.89%), reduced infarct size (−14.49%), mitigated fibrosis, and attenuated apoptosis through YAP nuclear translocation and Bcl‐2 upregulation, establishing that acellular biomaterials can harness intrinsic mechanical properties alone to reverse pathological remodeling via the YAP‐Bcl‐2 signaling axis [237]. Bio‐orthogonal double cross‐linked alginate‐gelatin hydrogels with tunable stiffness and stress relaxation via SPAAC and calcium crosslinking mimicked cardiac ECM viscoelasticity, with soft hydrogels promoting cardiac fibroblast spreading while stiffer hydrogels enhanced asymmetric elongation, maintaining high cell viability over 7 days [238]. A microfluidic‐fabricated anisotropic alginate‐gelatin methacrylate patch mimicked native myocardial anisotropy and guided directional cardiomyocyte polarization, significantly inhibiting ventricular remodeling and fibrosis while enhancing cardiac function recovery after MI (Figure 7A) [239]. A silk fibroin‐based heart valve leaflet fabricated through templated assembly achieved 46.9% β‐sheet crystals with 292% stacking strength enhancement and 1380% strength improvement over native silk, withstanding 60–180 mmHg cardiovascular pressure without failure and surpassing ISO 5840 requirements [240]. A silk fiber‐reinforced IPN hydrogel with controlled fiber orientation achieved tensile strengths up to 8.1 MPa and elastic moduli up to 28.2 MPa, with ultimate strains (46.5%–63.5%) matching native coronary arteries and cross‐plied configurations reproducing nonlinear strain‐stiffening and compliance (1.9%–2.1 %/100 mmHg) within human coronary ranges for vascular graft applications [241]. An injectable alginate hydrogel evaluated in mouse MI models revealed 342 differentially expressed mRNAs, with transcriptomic analysis identifying modulation of inflammation‐associated and chemokine‐related pathways, with validation confirming B2M modulation improving cardiac function, suggesting hydrogels directly alter cardiomyocyte inflammatory signaling beyond mechanical support [242].

**FIGURE 7:**
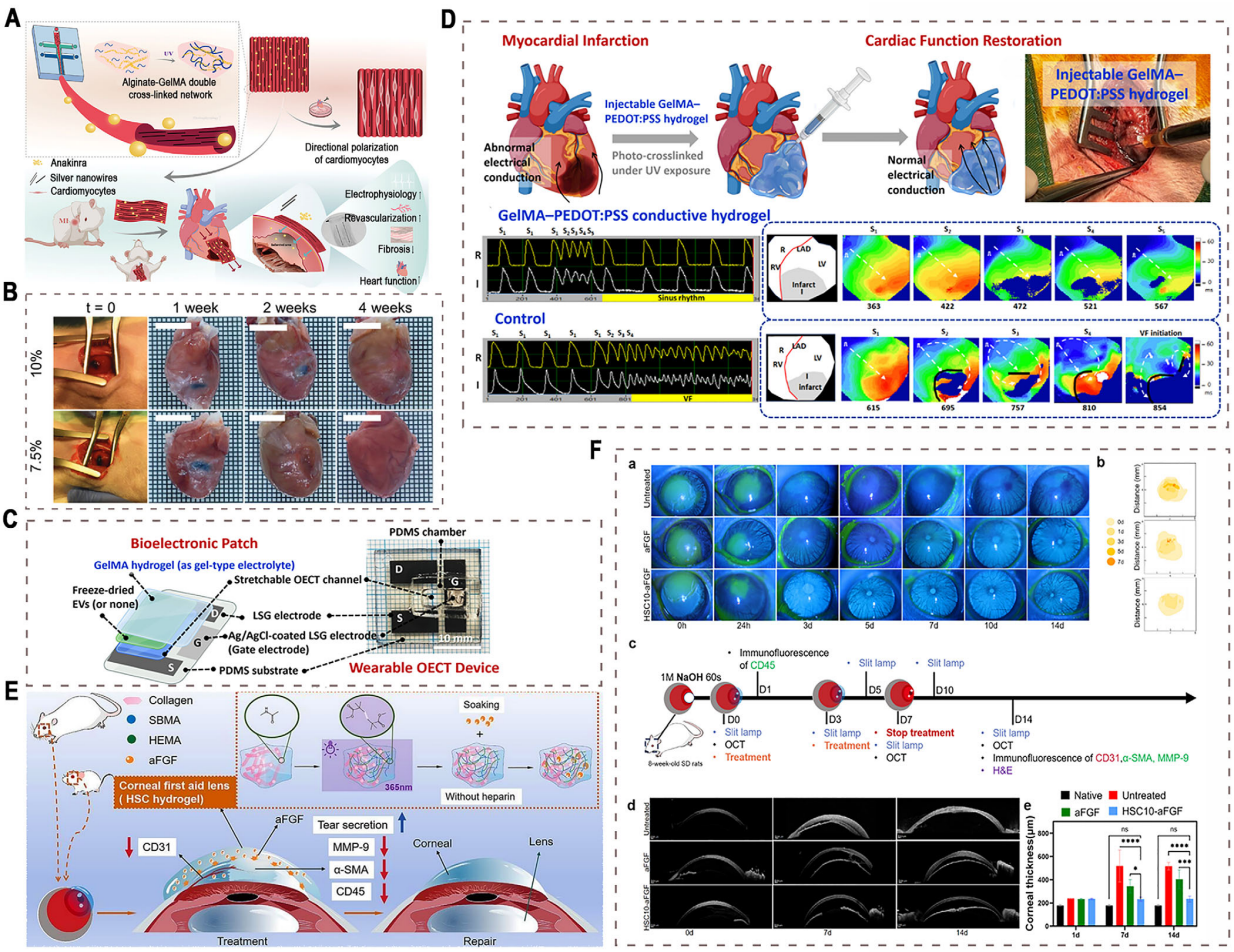
Multifunctional hydrogels application in cardiovascular and multitissue engineering. (A) Schematic illustration of the multifunctional anisotropic patches manufactured by microfluidic manipulation, and their application in guiding cardiomyocyte directional growth and treating myocardial infarction in rats. Reproduced with permission [239]. Copyright 2024, Wiley‐VCH. (B) Representative images of 10% (w/v) and 7.5% (w/v) HepK‐PEDOT hydrogels injected intramyocardially (*t* = 0), and explanted hearts after 1, 2, and 4 weeks postimplantation. Reproduced with permission [243]. Copyright 2025, Wiley‐VCH. (C) Schematic and photograph of an integrated bioelectronics patch featuring a wearable F10G0.5‐based OECT device encapsulated in UV‐crosslinked GelMA (G15P0), with PBS serving as gel‐type electrolyte, designed for controlled release of EVs and EP detection. (D) Injectable GelMA–PEDOT:PSS conductive hydrogel developed for MI treatment (White dashed arrows indicate the direction of wave propagation within the mapping field; black lines indicate functional conduction block. LAD, left anterior descending artery; LV, left ventricle; RV, right ventricle). Reproduced with permission [244]. Copyright 2025, Elsevier. (E) Schematic illustration of collagen‐based hydrogels loading aFGF as a contact lens for treating corneal injuries. (F) The effect of the HSC10‐aFGF hydrogel on corneal epithelial repair. Reproduced with permission [245]. Copyright 2025, Elsevier.

Conductive hydrogels have been developed to restore electrical coupling in infarcted myocardium while supporting cardiac cell function. An alginate/gelatin hydrogel with gold nanoparticles achieved superior conductivity (2.04 × 10^−^^4^ S cm^−^^1^), elevated Cx43 and CTnT expression in embryonic cardiac cells, and improved ejection fraction while reducing infarction size and fibrosis in rat MI models, promoting mechanical and electrical signal transmission between transplanted cells and surrounding tissue [246]. An injectable collagen‐PEDOT:PSS conductive hydrogel supported hiPSC‐cardiomyocyte maturation with near‐adult sarcomeric length, improved contractility, and enhanced calcium handling, with injection into infarcted mouse hearts decreasing ventricular tachycardia to healthy heart levels [247]. A GelMA‐PEDOT:PSS conductive hydrogel restored cardiac function and suppressed ventricular arrhythmias in a rabbit ischemia‐reperfusion model, while a bioelectronic patch integrating the hydrogel with stem cell‐derived EVs on an OECT device enabled controlled EV release and epinephrine biosensing for combined MI treatment and real‐time monitoring (Figure 7C,D) [244]. A TAlg/PEDOT@PMOF hydrogel with integrated nanofiller scavenged ROS via manganese porphyrin (mimicking antioxidant enzymes) while restoring electrical coupling via PEDOT, with cell‐adhesion peptides for synergistic function, improving cytoprotection, calcium transients, cardiac function, and reducing arrhythmia susceptibility [248]. A self‐doped sulfated glycosaminoglycan‐PEDOT hydrogel with a biodegradable GAG backbone and sulfate self‐doping achieved dual ionic/electronic conductivity with tunable stiffness and adhesive properties, facilitating electrical pacing of iPSC‐cardiomyocytes (Figure 7B) [243]. An injectable polyacrylamide/chitosan‐based PEDOT:PSS conductive hydrogel with irbesartan delivery achieved superior mechanical strength, conductivity, and wet‐adhesiveness, inhibiting inflammation and reducing infarct size in MIRI mouse models [249]. A tissue‐like optoelectronic biointerface integrating PEDOT:PSS achieved tissue‐matching mechanics (≈34 kPa), sevenfold charge injection enhancement, 156 mA W^−^^1^ responsivity, and >3‐year stability, enabling neuronal stimulation and photocontrol of cardiac myocyte beating frequency for wireless implants [250]. A polyacrylamide‐PEDOT:PSS hydrogel electrode achieved 5–68 kPa tensile strength, <3% hysteresis, and 99.6% ECG signal correlation with commercial electrodes, with high sensitivity across 0%–646% strain for wearable cardiac monitoring [251]. Micropatterned hydrogels with shear‐oriented cellulose nanocrystals produced anisotropic structural colors for visually monitoring cardiomyocyte beating, with microgrooves inducing cell alignment while autonomous beating caused hydrogel deformation and dynamic color shifts, enabling noninvasive real‐time monitoring of cardiac tissue under pharmaceutical and electrical stimulation [252].

These studies demonstrate that cardiovascular tissue engineering increasingly depends on integrating mechanical matching, electrical conductivity, and phase‐specific therapeutic delivery within hydrogel platforms.

### 6.5 Emerging and Multitissue Applications

Hydrogels are finding expanding applications across diverse tissue systems beyond the well‐established contexts of wound healing, musculoskeletal repair, neural regeneration, and cardiovascular engineering. Liver and pancreatic regeneration benefit from hydrogels that mimic the hepatocyte and islet microenvironment, addressing the high metabolic demands and secretory functions of these organs [253, 254, 255]. Kidney regeneration hydrogels must accommodate complex filtration and transport functions [256], while muscle regeneration hydrogels deliver myoblasts, mesenchymal stem cells, or growth factors to injured or dystrophic muscle [257]. Ocular regeneration hydrogels address corneal defects, retinal degeneration, and glaucoma through targeted delivery and controlled release, with the unique anatomical and immunological characteristics of the eye requiring specialized formulations (Table 2) [258, 259]. Dental and periodontal hydrogels deliver cells and factors to regenerate dental pulp and periodontal tissues [128]. Multitissue interface regeneration, including bone‐tendon and bone‐ligament interfaces, demands hydrogels with spatially resolved properties that address distinct tissue requirements [260, 261].

**TABLE 2:** Therapeutic applications and design requirements of multifunctional hydrogels across tissue types.

Tissue Type	Clinical need	Hydrogel design requirements	Therapeutic strategies	Key bioactive cargos	Preclinical models	Primary clinical challenges	Ref.
Skin/wound healing	Acute and chronic wounds (diabetic ulcers, pressure ulcers, burns)	Moisture management, exudate absorption, antimicrobial activity, conformability	Growth factor delivery, stem cell/EV delivery, antimicrobial release, immunomodulation	VEGF, PDGF, EGF, FGF, antimicrobial peptides, MSC‐EVs, antibiotics	Diabetic mouse (db/db, STZ), porcine wound models	Infection control, exudate management, offloading compliance	[96, 167, 171, 173, 175, 176, 180]
Bone	Critical‐sized defects, osteoporosis, fractures, osteomyelitis	Mechanical strength (MPa range), osteoconductivity, porosity for vascularization	BMP‐2 delivery, MSC delivery, osteoinductive nanoparticles, immune modulation	BMP‐2, BMP‐7, TGF‐β, hydroxyapatite, calcium phosphate, MSCs	Rat calvarial, rabbit femoral, sheep models	BMP complications (cancer, ectopic bone), load‐bearing needs	[188, 189, 192, 196, 201, 202]
Cartilage	Osteoarthritis, traumatic cartilage defects	Compressive strength, low friction, chondroinductive, injectable	Chondrocyte/MSC delivery, ECM component delivery, growth factor delivery	TGF‐β, IGF‐1, chondroitin sulfate, dECM, MSCs	Rat, rabbit, porcine cartilage defect models	Avascular nature, load‐bearing, mechanical matching	[191, 203, 206, 207, 208]
Nervous system	Spinal cord injury, traumatic brain injury, stroke, peripheral nerve injury	Guidance cues, soft mechanics, neurotrophic factor delivery, immune modulation	Neurotrophic factor delivery, stem cell delivery, axonal guidance, immunomodulation	NGF, BDNF, GDNF, NT‐3, MSCs, NSCs	Rat SCI, mouse TBI, peripheral nerve gap models	Blood–brain barrier, inhibitory environment, complex anatomy	[214, 215, 217, 218, 219, 224]
Cardiovascular	Myocardial infarction, heart failure, peripheral artery disease	Compliance matching, conductivity (cardiac), angiogenic, injectable	Stem cell delivery, growth factor delivery, conductive materials, immunomodulation	VEGF, HGF, IGF‐1, MSCs, iPSC‐CMs, conductive polymers	Rat MI, porcine MI, hindlimb ischemia models	Cardiac function, mechanical matching, scalability	[228, 229, 232, 233, 234, 235, 238]
Liver	Liver failure, cirrhosis, hepatic resection	High metabolic demand, vascularization, hepatocyte support	Stem cell/secretome delivery, growth factor delivery, ECM mimicry	MSC‐CM, HGF, EGF, hepatocyte growth factors	Partial hepatectomy models, cirrhosis models	Vascularization, metabolic demand, hepatocyte polarity	[253, 254]
Kidney	Diabetic nephropathy, chronic kidney disease, acute kidney injury	Renal niche mimicry, controlled release	EV delivery, anti‐fibrotic delivery, stem cell delivery	MSC‐EVs, anti‐inflammatory agents	Diabetic nephropathy mouse models	Complex renal architecture, filtration functions	[256]
Muscle	Muscular dystrophy, volumetric muscle loss, ischemic injury	Mechanically supportive, angiogenic, myogenic	Myoblast/MSC delivery, growth factor delivery, electrical stimulation	VEGF, IGF‐1, HGF, myoblasts, MSCs	Muscle injury, hindlimb ischemia models	Vascularization, innervation, functional integration	[257, 262, 263]
Ocular	Corneal defects, retinal degeneration, glaucoma	Optical transparency, conformability, sustained release	Drug delivery, cell delivery, neuroprotection	Anti‐VEGF, neurotrophic factors, stem cells	Corneal injury, retinal degeneration models	Drug delivery to posterior segment, toxicity, immunogenicity	[245, 258, 259, 264, 265, 266, 267]

Abbreviations: BDNF, brain‐derived neurotrophic factor; BMP, bone morphogenetic protein; dECM, decellularized extracellular matrix; EGF, epidermal growth factor; EV, extracellular vesicle; FGF, fibroblast growth factor; GDNF, glial cell line‐derived neurotrophic factor; HGF, hepatocyte growth factor; IGF‐1, insulin‐like growth factor 1; iPSC‐CM, induced pluripotent stem cell‐derived cardiomyocyte; MI, myocardial infarction; MSC, mesenchymal stem cell; NGF, nerve growth factor; NT‐3, neurotrophin‐3; PDGF, platelet‐derived growth factor; SCI, spinal cord injury; STZ, streptozotocin; TBI, traumatic brain injury; TGF‐β, transforming growth factor beta; VEGF, vascular endothelial growth factor.

Recent advances in liver tissue engineering have demonstrated the potential of hybrid hydrogel scaffolds for hepatic cell culture and regeneration. A degradable PVA‐urea oligomer hybrid hydrogel reinforced with egg white, gelatin, collagen, and chitosan achieved high tensile strength, approximately 70% degradation after 14 days, and supported hepatocyte and fibroblast growth and proliferation over 5 days, demonstrating potential for liver tissue engineering [268]. A porcine liver‐derived ECM hydrogel containing 34.9% protein enhanced liver sinusoidal endothelial cell and hepatocyte viability, reduced LSEC dedifferentiation, and maintained hepatocyte function longer than traditional culture through bioactive collagens and mechanical/microarchitectural properties [269]. For kidney applications, a collagen‐poly(acrylic acid)‐quaternized chitosan hydrogel patch achieved 172.60 kPa tensile strength, 51.28 kPa wet adhesion surpassing clinical benchmarks, and promoted rapid hemostasis and orderly renal tubule regeneration in a rat partial nephrectomy model [270]. Tunable GelMA hydrogels with stiffness matching adult human kidney (≈5000–10 000 Pa) improved hiPSC‐derived kidney organoid podocyte maturation and upregulated renal vesicle‐associated genes compared with softer matrices, while enabling diabetic kidney disease modeling, with softer matrices reducing pSMAD3 and α‐SMA expression [271]. A microalgae‐based oral hydrogel (BBR‐CV@ALG) enabled prolonged gastrointestinal lead biosorption, removing lead from the liver, kidney, and bone, while berberine reduced oxidative tissue damage and repaired the intestinal barrier, offering a biodegradable, low‐cost approach for lead poisoning treatment [272].

Ocular tissue engineering has similarly benefited from hydrogel platforms designed to replicate the unique structural and optical properties of the cornea. A 3D bioprinted GelMA/collagen hydrogel with convex corneal architecture suppressed keratocyte‐to‐fibroblast transformation and preserved keratocyte phenotype, effectively reducing corneal fibrosis in vivo [264]. An electrospun gelatin‐HA hydrogel with dual crosslinking achieved 98% optical transmittance and 3.141 MPa tensile modulus, closely mimicking native corneal properties with high permeability and controlled degradability for corneal transplantation [265]. A visible light‐curable collagen‐PEGDA‐DOPA bioadhesive achieved 783.6 kPa adhesive strength and 53.7 kPa shear strength, surpassing commercial eye adhesives with >100% keratocyte survival for corneal perforation repair [266]. A glycidyl methacrylate‐grafted gelatin hydrogel (GELGYM) achieved 1.95 MPa tensile stress, 80% compressive strain, and super adhesion to cornea and multiple tissues, supporting cell adhesion, proliferation, and migration in two‐dimensional (2D) and 3D cultures [267]. A collagen‐based contact lens with dual‐step orthogonal crosslinking enabled controlled aFGF release without heparin, demonstrating anti‐inflammatory and antifibrotic effects for corneal alkali burn treatment as a corneal first aid lens for self‐management and postoperative care (Figure 7E,F) [245].

Muscle tissue engineering and multitissue applications have also advanced through the development of mechanically reinforced and bioelectronic hydrogel platforms. A MXene/PCL nanofiber‐hydrogel composite scaffold (MPHP) enhanced myogenic differentiation (>3.9‐fold MyoD, >2.9‐fold MyoG), promoted M2 macrophage polarization, angiogenesis, and neuromuscular reinnervation, achieving a 38% increase in exercise tolerance in volumetric muscle loss models [262]. An alginate‐gelatin‐fibrinogen hydrogel with shear‐thinning behavior, good printability, and tunable modulus/porosity via dual crosslinking supported C2C12 myoblast growth and enabled 3D bioprinting of complex muscle constructs and biohybrid robotic systems [263]. A reusable PEDOT:PSS‐PDA‐PAM hydrogel achieved 5.57 S m^−^^1^ conductivity, 2.7 kPa modulus, 46.45 kPa adhesion with 87% retention after 200 cycles, and 94% self‐healing efficiency, with electromyography monitoring patches demonstrating 30.05 dB SNR (vs. 12.06 dB commercial) for long‐term monitoring [273]. A PEDOT:PSS‐poly(acrylic acid) hydrogel supercapacitor achieved 97.81% capacity retention after 10,000 cycles, 233% tensile strain, and 6.42 kPa tissue adhesiveness with excellent biocompatibility in mice for implantable microintelligent medical devices [274]. An injectable photocrosslinked methacrylated silk fibroin‐hyaluronic acid hydrogel with enhanced mechanical properties supported human dental pulp stem cell growth, proliferation, and osteogenic differentiation for dental pulp tissue engineering [275].

Overall, these emerging applications demonstrate that multifunctional hydrogels are increasingly being adapted to address the unique biological, mechanical, and anatomical requirements of diverse tissue systems. However, the translation of these platforms from proof‐of‐concept studies to clinical applications faces common challenges, including the need for tissue‐specific mechanical matching, long‐term biocompatibility, and scalable manufacturing.

## 7 Preclinical Models and Clinical Translation

Despite impressive preclinical advancements, the translation of multifunctional hydrogels from laboratory research to clinical practice faces substantial biological, regulatory, and manufacturing hurdles that must be systematically addressed. This section examines the preclinical animal models used to evaluate hydrogel‐based therapies, the regulatory pathways governing product classification and approval, the critical pain points limiting clinical translation, and the current landscape of clinical trials, providing a realistic assessment of the gap between bench innovation and bedside implementation.

### 7.1 Preclinical Animal Models

Preclinical evaluation of hydrogel‐based regenerative therapies typically proceeds through in vitro characterization followed by in vivo assessment in animal models. The selection of appropriate animal models is critical for predicting clinical performance and establishing therapeutic efficacy.

#### 7.1.1 Small Animal Models

Rodents, particularly mice and rats, remain the most widely used preclinical models due to their cost‐effectiveness, well‐characterized genetics, and availability of immunological reagents [276]. Wound healing studies in rodents can assess re‐epithelialization, angiogenesis, and granulation tissue formation, though the role of contraction (predominant in rodents) versus re‐epithelialization (predominant in humans) must be considered. Subcutaneous implantation models assess biocompatibility and degradation, providing initial data on host response to hydrogel materials [277]. These models can evaluate inflammatory responses, fibrotic encapsulation, and degradation kinetics, though the subcutaneous environment differs from target tissue sites. Bone defect models in rats and rabbits enable assessment of hydrogel‐based bone regeneration, with critical‐sized defects providing a stringent test of regenerative capacity [278]. Immunocompromised mouse models enable the assessment of human cell and tissue components, though the lack of a competent immune system limits evaluation of immunological responses.

#### 7.1.2 Large Animal Models

Large animal models, including pigs, sheep, dogs, and nonhuman primates, provide more clinically relevant assessments due to similarities in size, physiology, and immunology to humans [279]. Pig models are particularly valuable for wound healing due to similarities in skin structure and healing physiology. Sheep models are widely used for bone and cartilage regeneration, while canine models have been used for cardiovascular and spinal applications [280]. The translational relevance of large animal models must be balanced against ethical considerations, cost, and availability of appropriate reagents and imaging modalities [279]. The use of large animals is typically justified for late‐stage preclinical evaluation after promising results in rodent models.

#### 7.1.3 Diabetic Wound Models

Diabetic wound models, including db/db mice, streptozotocin‐induced diabetic rats, and diabetic pigs, are essential for evaluating chronic wound therapies [281]. These models mimic aspects of human diabetic wounds, including delayed healing, impaired angiogenesis, and altered inflammatory responses. However, no animal model fully recapitulates the complex pathophysiology of human diabetic foot ulcers, necessitating careful interpretation of results. The use of diabetic wound models should consider factors including glycemic control, wound infection, and mechanical offloading that are relevant to human diabetic wound management. Clinically relevant wound sizes and locations should be used to enable translation.

### 7.2 Regulatory Pathways

The regulatory pathway for hydrogel‐based therapeutic products depends on product classification, which is determined by primary mechanism of action. Hydrogels may be classified as medical devices, drugs, biological products, or combination products, each with distinct regulatory requirements [282]. Medical device classification applies to hydrogels that achieve their primary therapeutic effect through physical mechanisms, including wound dressings, surgical sealants, and tissue bulking agents. These products must demonstrate safety and effectiveness through bench testing, animal studies, and clinical trials, with the level of evidence required determined by risk classification [283]. The 510(k) pathway enables market clearance based on substantial equivalence to a legally marketed predicate device, while the Premarket Approval (PMA) pathway requires independent demonstration of safety and effectiveness [284].

Drug classification applies to hydrogels that achieve their primary therapeutic effect through pharmacological mechanisms, including those incorporating therapeutic agents with release as a critical feature [285]. These products require extensive preclinical and clinical evidence of safety and efficacy, with New Drug Application (NDA) submission. The inclusion of proteins, peptides, or nucleic acids may classify the hydrogel as a biological product, requiring a Biologics License Application (BLA). Combination product classification applies to products incorporating both device and drug or biological components, with the primary mode of action determining the lead regulatory center [286]. Hydrogel delivery systems for growth factors, cells, or gene therapy typically fall into this category, requiring coordinated review by multiple FDA centers. Early regulatory engagement through pre‐IND or pre‐Submission meetings is strongly recommended for novel hydrogel products (Figure 8).

**FIGURE 8:**
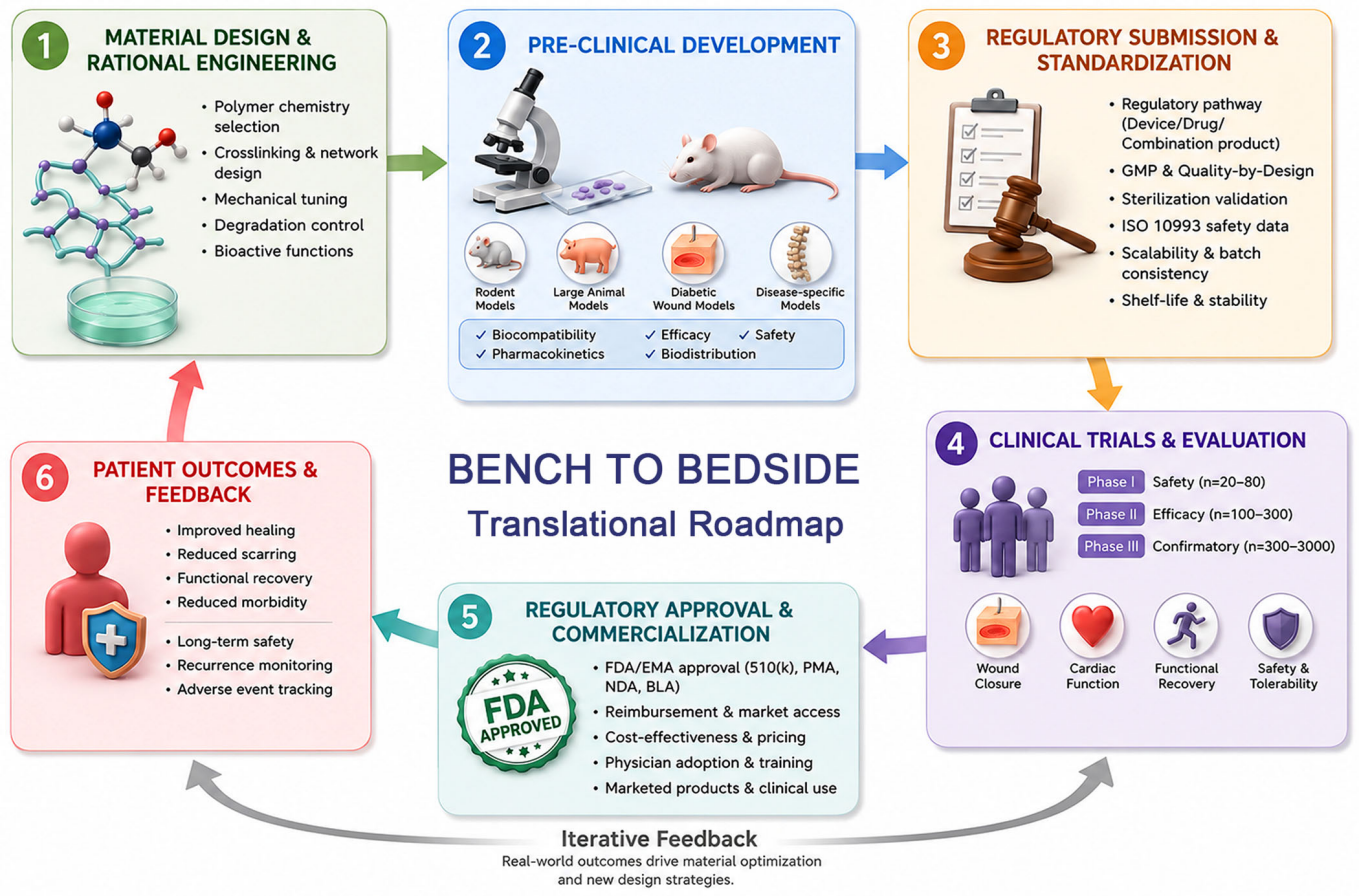
Bench‐to‐bedside translational roadmap for multifunctional hydrogels. This schematic illustrates the six‐stage translational pathway from material design and engineering through preclinical development, regulatory submission, clinical trials, regulatory approval, and patient outcomes, with iterative feedback loops connecting clinical results to material optimization. Critical translational barriers (including scalability, sterilization, regulatory classification, and cost‐effectiveness) are highlighted across the pathway, emphasizing that successful clinical translation requires balancing biological complexity with manufacturing feasibility and regulatory practicality.

### 7.3 Clinical Translation Pain Points

Despite substantial progress, the clinical translation of multifunctional hydrogels remains limited by challenges beyond biological efficacy. Many systems demonstrate promising results in small‐animal models but face difficulties related to scalable manufacturing, reproducible quality control, sterilization, storage stability, and regulatory approval. Increasing functional complexity may improve therapeutic performance but simultaneously complicate manufacturing and clinical evaluation. Therefore, future development should prioritize clinically realistic multifunctionality, where selected functions provide clear therapeutic advantages while maintaining manufacturability and regulatory feasibility [287].

#### 7.3.1 Scalability and Batch‐to‐Batch Consistency

Hydrogel manufacturing scalability presents significant challenges, particularly for natural polymer‐derived systems where batch‐to‐batch variability is inherent. Standardized sourcing of raw materials, rigorous quality control, and reproducible processing conditions are essential for commercial‐scale production [288]. The integration of automation and process analytical technology can improve batch consistency. Synthetic polymer systems offer better batch‐to‐batch consistency, though functionalization and crosslinking introduce variability that must be controlled. The complexity of multifunctional hydrogels, incorporating multiple components and active agents, further challenges manufacturing consistency and quality control.

#### 7.3.2 Sterilization Compatibility

Hydrogel sterilization compatibility must be considered early in product development. Terminal sterilization methods, including ethylene oxide, gamma irradiation, and autoclaving, may degrade hydrogel components or alter crosslinking [289, 290, 291]. Irradiation sterilization may also cause allergies or the production of toxic substances. Filtration sterilization is suitable for liquid precursors but incompatible with gelled products. Aseptic processing, while maintaining product integrity, adds cost and complexity to manufacturing. The selection of sterilization method must account for hydrogel composition, architecture, and functional components [292]. Sterilization validation studies should assess product functionality poststerilization, including mechanical properties, release kinetics, and biological performance. Sterile packaging and shelf‐life stability are additional considerations for commercial products.

#### 7.3.3 In Situ Crosslinking Safety

In situ crosslinking presents safety considerations including photoinitiator toxicity, light exposure effects, and heat generation [125]. Photoinitiators must be selected for cytocompatibility and used at the minimum effective concentration. Light exposure parameters, including wavelength, intensity, and duration, must be optimized for patient safety while achieving sufficient crosslinking. Reactive species generated during crosslinking may cause cellular damage, necessitating careful optimization and characterization. The clinical environment presents additional challenges, including variable wound geometry, presence of blood and exudate, and constraints on light delivery.

#### 7.3.4 Cost‐Effectiveness and Reimbursement

The cost‐effectiveness of hydrogel‐based therapies must be demonstrated relative to existing standards of care. The development of advanced hydrogel products involves significant research, development, and clinical trial costs that must be justified by therapeutic benefit. Demonstration of improved outcomes, reduced complications, shorter hospital stays, and decreased overall healthcare costs is essential for reimbursement approval. Reimbursement pathways differ between countries and healthcare systems, with product classification significantly influencing reimbursement processes. Engagement with payers and health technology assessment bodies during product development can inform clinical trial design and demonstrate economic value.

### 7.4 Clinical Trial Landscape

The clinical translation of hydrogel‐based regenerative therapies has progressed significantly, with numerous products in clinical trials and several receiving regulatory approvals [9]. Early clinical trials have focused on wound healing, where the accessible site and established clinical endpoints facilitate evaluation.

Wound healing hydrogels with regulatory approval include alginate, hydrocolloid, and hydrogel dressings for diabetic ulcers, venous ulcers, and pressure ulcers [171]. These products primarily provide moisture management and wound protection, with limited active therapeutic components. Recent regulatory developments include FDA 510(k) clearance for DermiSphere Hydrogel Dermal Regeneration Template (2025), a collagen‐based hydrogel designed for tissue integration and revascularization [293], and ECMT‐100 WMD, a flowable ECM hydrogel cleared for wound management (Table 3) [294].

**TABLE 3:** Clinical trials of hydrogel‐based therapies in regenerative medicine.

Hydrogel systems	Indication	Trial identifier	Phase	Status	Study design	Primary endpoint	Key findings/status	Ref.
DermiSphere hydrogel dermal regeneration template	Full‐thickness wounds	FDA 510(k) Clearance (2025)	Regulatory Approval	Approved	Not applicable	Not applicable	FDA 510(k) clearance for collagen‐based hydrogel designed for tissue integration and revascularization	[293]
ECMT‐100 WMD (flowable ECM hydrogel)	Wound management	FDA 510(k) Clearance (2026)	Regulatory Approval	Approved	Not applicable	Not applicable	FDA 510(k) clearance (K253521) for wound management	[294]
rhGM‐CSF + hydrogel (Repogel‐01)	Venous leg ulcers	NCT04823962	Phase II	Active, not recruiting	Randomized, placebo‐controlled, double‐blind	Wound healing acceleration	Assessing safety and efficacy; not yet reported	[295]
Acellular dermal matrix (ADM) hydrogel	Chronic traumatic wounds	NCT06978569	Not applicable	Not yet recruiting	Randomized, outcome assessor‐blinded (vs. alginate dressing)	Wound surface area reduction at Week 12	Comparative efficacy trial; not yet available	[296]
HERADERM hydrogel wound dressing	Cesarean section surgical site wounds	NCT06558240	Not applicable	Completed (Jul 2024 – Estimated Jul 2025)	Observational, prospective, single‐arm	REEDA scale (wound healing assessment)	Healing outcomes assessed using REEDA scale; not yet published	[297]
Cartistem (hUCB‐MSCs + HA hydrogel)	Osteoarthritis/cartilage repair	NCT03754465	Phase II	Completed	Interventional, parallel assignment	Complete wound repair within 12 weeks	Improved clinical outcomes in osteoarthritic patients	[298]
ChonDux hydrogel (chondroitin sulfate‐PEG)	Full‐thickness articular cartilage defects (knee)	NCT01110070	Not applicable	Completed	Assessor‐blinded, single‐arm (with microfracture)	Defect volume fill at 6 months (MRI)	Stable repair of full‐layer articular cartilage defects in 18 patients	[299]
Albumin‐hyaluronic acid hydrogel	Knee cartilage defects	NCT03319797	Phase III	Completed	Prospective, multicenter, single‐arm	KOOS responder rate at 2 years	93% KOOS responder rate at 2 years posttreatment	[300]
Peptide hydrogel (PeriHeal)	Periodontal regeneration	NCT07581613	Phase I/II	Not yet recruiting (Start: Nov 2026)	Randomized, parallel‐arm (vs. hyaluronic acid hydrogel)	Clinical attachment level (CAL) change at 6 and 12 months	Currently being investigated in a multicenter randomized controlled trial	[301]

Abbreviations: ADM, acellular dermal matrix; CAL, clinical attachment level; ECM, extracellular matrix; FDA, Food and Drug Administration; GM‐CSF, granulocyte‐macrophage colony‐stimulating factor; HA, hyaluronic acid; hUCB‐MSCs, human umbilical cord blood‐derived mesenchymal stem cells; KOOS, Knee Injury and Osteoarthritis Outcome Score; MRI, magnetic resonance imaging; PEG, polyethylene glycol; REEDA, redness, edema, ecchymosis, discharge, approximation; WMD, wound management device.

Advanced products incorporating antimicrobial agents, growth factors, or cells are in clinical development. For example, a Phase II clinical trial (NCT04823962) is evaluating recombinant human GM‐CSF incorporated into a hydrogel for venous leg ulcers, with the objective of assessing safety and efficacy in accelerating wound healing [295]. A comparative efficacy trial (NCT06978569) is investigating acellular dermal matrix hydrogel versus alginate dressings in chronic traumatic wounds [296]. Additionally, HERADERM Hydrogel Wound Dressing (NCT06558240) has been evaluated for cesarean section surgical site wound care, with studies assessing healing outcomes using the REEDA scale [297].

Cell delivery hydrogels have progressed to clinical trials for cartilage repair and wound healing. For instance, Cartistem, a hyaluronic acid hydrogel complex with allogeneic human umbilical cord blood‐derived mesenchymal stem cells (NCT03754465), has demonstrated improved clinical outcomes in osteoarthritic patients [298]. ChonDux Hydrogel (a chondroitin sulfate‐PEG hydrogel) (NCT01110070) has shown stable repair of full‐layer articular cartilage defects in 18 patients [299], while an albumin‐hyaluronic acid hydrogel in a Phase III trial achieved 93% KOOS responder rates at two years posttreatment (NCT03319797) [300]. A peptide hydrogel (NCT07581613) is currently being investigated for periodontal regeneration in a multicenter randomized controlled trial [301].

Growth factor delivery hydrogels have also been evaluated in clinical trials for wound healing and bone regeneration. The challenges of achieving sustained, localized growth factor delivery have been partially addressed through advanced hydrogel formulation, though further optimization is needed for consistent clinical outcomes. A systematic review of clinical trials on hydrogel dressings for chronic wounds concluded that cellulose‐based hydrogels were predominantly used, with findings suggesting reduced healing time and increased wound‐size reduction compared with standard care [302].

## 8 Translational Roadmap and Future Perspectives

The future of multifunctional hydrogels will be shaped by the convergence of predictive design tools, advanced fabrication technologies, and deeper biological understanding that together enable the development of smarter, more adaptable regenerative platforms. This section outlines the translational roadmap for next‐generation hydrogel systems, discussing rational design for predictive performance, advanced manufacturing and biofabrication, smart and adaptive behaviors, multitherapeutic integration, regulatory harmonization, and emerging directions, while also critically examining unresolved controversies and questions that must be addressed to accelerate clinical adoption.

### 8.1 Rational Design for Predictive Performance

The future of multifunctional hydrogels depends on the establishment of rational design principles that enable predictive engineering of material performance. Computational modeling, including molecular dynamics simulations, finite element analysis, and machine learning approaches, can accelerate material development and reduce reliance on empirical testing [303]. Predictive models linking hydrogel composition, processing, and structure to mechanical properties, degradation kinetics, and biological performance can guide rational design decisions [304]. The integration of quantitative structure–property relationships with biological response data can identify optimal material formulations for specific therapeutic applications. The establishment of standardized characterization protocols would facilitate comparison between studies and accelerate translation. Consensus standards for hydrogel mechanical testing, degradation assessment, and biocompatibility evaluation would reduce barriers to regulatory approval.

### 8.2 Advanced Manufacturing and Biofabrication

Additive manufacturing technologies, including 3D printing, bioprinting, and digital light processing, enable the fabrication of complex hydrogel constructs with precise spatial control over architecture and composition [305]. These technologies can produce patient‐specific constructs, complex internal architectures, and spatially resolved mechanical and biochemical properties. The integration of multiple material types in single constructs, including hydrogels with different properties, enables the recapitulation of tissue interfaces and gradients. Multimaterial bioprinting can fabricate osteochondral constructs with graded mechanical properties and biochemical composition, nerve guidance conduits with supportive glial cues, and vascular grafts with luminal endothelialization [192]. The scalability and throughput of additive manufacturing must be improved for clinical translation, with techniques including high‐speed printing and continuous printing addressing manufacturing efficiency. The development of standardized bioinks and print parameters would facilitate regulatory approval and technology adoption.

### 8.3 Smart and Adaptive Hydrogel Systems

The next generation of hydrogel systems will incorporate adaptive behaviors that respond to physiological cues, enabling personalized and temporally coordinated therapeutic delivery. Smart hydrogels that sense and respond to wound status, disease progression, or tissue regeneration can adjust therapeutic delivery dynamically, optimizing treatment over time.

Self‐regulating hydrogels that release therapeutics in response to specific signals, including pH, temperature, enzymes, and glucose, can provide on‐demand therapy that matches physiological needs. Self‐healing hydrogels that repair mechanical damage can maintain therapeutic function in dynamic tissue environments [306]. Bioelectronic hydrogels that integrate sensors and actuators enable closed‐loop therapeutic systems. The integration of multiple adaptive behaviors within a single hydrogel platform can achieve sophisticated therapeutic strategies, including sequential delivery corresponding to different phases of wound healing or tissue regeneration. The development of hydrogels that evolve their properties over time, mimicking the dynamic changes in native tissue, represents a frontier in regenerative material design.

### 8.4 Multitherapeutic Integration Strategies

The therapeutic demands of complex tissue injuries and chronic diseases often require multitargeted interventions that cannot be achieved through single‐agent delivery. Multifunctional hydrogels that integrate multiple therapeutic functionalities within a single platform can address this complex pathophysiology.

Sequential therapeutic delivery, where different agents are released at specific times corresponding to physiological needs, can coordinate regeneration across multiple phases [307]. Early release of anti‐inflammatory agents, followed by angiogenic factors, and then matrix‐stabilizing agents, can support the progression through wound‐healing phases. The development of hydrogels with programmable, multiphasic release kinetics requires careful control over multiple independent release mechanisms. Spatiotemporally coordinated delivery, where therapeutic agents are released at specific times and locations, can achieve synergistic effects that exceed individual contributions [49]. The localized delivery of immunomodulatory and angiogenic factors, coordinated to specific regeneration phases, can create positive feedback loops that accelerate and enhance regeneration.

The integration of molecular, cellular, and material functionalities within a single hydrogel platform can achieve comprehensive therapeutic strategies that address multiple aspects of tissue injury and regeneration [9]. Molecular therapies, including drugs, growth factors, and nucleic acids, can modulate cellular behavior and signaling. Cellular therapies can provide dynamic, responsive sources of therapeutic factors. Material properties can provide structural support and environmental cues.

### 8.5 Regulatory Harmonization and Standardization

The advancement of multifunctional hydrogels to clinical application demands regulatory harmonization and standardization [282]. The development of consensus standards for hydrogel characterization, including mechanical testing, degradation assessment, and biocompatibility evaluation, would facilitate product development and regulatory approval. Regulatory science research, including the development of alternatives to animal testing and predictive models for human performance, can accelerate translation while maintaining safety standards [277, 308]. International harmonization of regulatory requirements would reduce barriers to global product development and market access. Early regulatory engagement, including pre‐IND meetings and device development guidance, can identify critical data requirements and streamline development pathways. The formation of public–private partnerships for regulatory science can support the development of standards and tools specific to hydrogel products.

### 8.6 Clinical and Commercial Translation

The clinical translation of multifunctional hydrogels requires integration across multiple domains, including material development, manufacturing, clinical research, and commercialization [309]. A successful translation strategy must address the practical requirements of clinical application, including user training, reimbursement, and adoption into clinical practice. Clinical trial design must account for the unique characteristics of hydrogel products, including the learning curve for application, the potential for off‐target effects, and the importance of patient stratification [310]. Appropriate clinical endpoints must be selected to demonstrate meaningful therapeutic benefit, considering both objective outcomes and patient‐reported measures. Surrogate endpoints may be appropriate for early‐phase trials, with definitive endpoints required for product approval.

Commercialization strategies must address manufacturing scale‐up, regulatory approval, market access, and reimbursement. The identification of target patient populations and clinical indications with unmet need can inform product development and market positioning. Partnership with established medical device or pharmaceutical companies can provide access to manufacturing and commercial infrastructure. The development of business models for multifunctional hydrogel products must account for research and development costs, manufacturing costs, clinical trial costs, and market size. Value‐based pricing, where product price is linked to demonstrated therapeutic benefit, can support reimbursement and market adoption.

### 8.7 Future Outlook and Emerging Directions

The future development of multifunctional hydrogels will be shaped by the integration of advanced technologies that enable predictive design, adaptive regulation, and personalized regenerative therapies. A major transition is expected from empirical optimization toward data‐driven hydrogel engineering, where artificial intelligence, computational modeling, high‐throughput screening, and quantitative structure–property–function relationships facilitate the rational prediction of material performance. In this context, AI‐driven modeling approaches using databases of over 1,200 bioprinting tests have demonstrated high predictive accuracy for printing properties (specificity 88.4%, sensitivity 86.2%), enabling in silico optimization without trial‐and‐error testing and significantly accelerating personalized medicine and tissue engineering applications [311]. Similarly, advanced constitutive models incorporating nonaffine deformation of polymer networks have improved the prediction of hydrogel mechanical behavior under extreme deformation, providing a theoretical foundation for rational design of hydrogel‐based devices [312]. AI algorithms have also been employed to design and optimize crosslinked hydrogel microneedle patches incorporating 5‐fluorouracil‐loaded flexible PEGylated liposomes for nonmelanoma skin cancer treatment, achieving optimal particle size (36.23 ± 2.42 nm), high skin permeation (41.78 ± 4.26%), and effective cancer inhibition with apoptosis promotion [313].

Advanced manufacturing approaches are rapidly expanding the capabilities of hydrogel fabrication. Four‐dimensional printing has emerged as a transformative technology, enabling hydrogels to undergo programmable shape transformations over time in response to physiological stimuli. A thermoresponsive 4D‐printed hydrogel ink composed of poly(N‐isopropylacrylamide), alginate, and elastic biosourced nanocellulose fibers achieved anisotropic shape morphing at body temperature (≥36°C) with tensile strength of 150 kPa and toughness of 83 kJ m^−^^3^, demonstrating potential for tissue engineering and soft robotic applications [314]. A 4D‐printed multifunctional conductive hydrogel based on alginate incorporating acid‐functionalized carbon nanotubes achieved electrical conductivity of 6.6 ± 0.5 mS cm^−^^1^, enabling strain sensing of human motions, while programmable shape changes via differential swelling yielded nerve guidance conduits with intricate designs (single, multichannel, and bifurcated configurations) accurately predicted by finite element analysis, with enhanced neural cell proliferation and sutureless peripheral nerve regeneration demonstrated in rat models [315]. A DLP‐printed 4D hydrogel with thermo‐responsive NIPAm mainchains for body‐temperature‐activated contraction, PEGDA‐dopamine crosslinkers for adhesion and degradability, and curcumin‐loaded micelles for antibacterial activity accelerated diabetic wound healing in MRSA‐infected rat models through combined mechanobiological contraction and inflammation regulation [316]. A programmable ceramic precursor system based on synergistic reinforcement of phase‐separating hydrogels and shape memory polymers enabled high‐precision stereolithographic 4D printing with programmable recovery onset controlled by composition, programming time, and thickness, with layered printing strategies enabling autonomous reversible deformation [317]. A high‐swelling composite hydrogel (SwellMA) combining gelatin methacryloyl and sodium polyacrylate achieved swelling capacity exceeding 500% of its original area with a 100‐fold water weight increase and cyclic on‐demand swelling‐shrinking upon ionic strength changes, enabling advanced 4D bioprinting with superior cytocompatibility and printing fidelity [318]. Ion‐transfer printing technology enabled reprogrammable 4D tissue engineering scaffolds with tunable crosslinking gradients, allowing “3D‐to‐3D” shape conversions and shape reprogrammability for engineered cartilage‐like constructs [319]. A single‐component jammed micro‐flake hydrogel system was developed as a cell‐laden bioink for 4D bioprinting with shear‐thinning, self‐healing properties and high cell viability, enabling 4D cartilage‐like tissue formation [320]. A next‐generation embedded bioprinting platform (REFRESH) using custom‐engineered PEG‐gelatin microgel baths achieved high‐fidelity fabrication of anatomically relevant tissue constructs from decellularized ECM hydrogels, recreating tracheal architecture and trifurcated bronchial structures with primary cells [321]. A dissolvable temporary barrier strategy enabled flexible hydrogel patterning in organ‐on‐a‐chip models, generating 3D vascular networks through vasculogenesis and angiogenesis in vascularized microtumor models for multiorgan chip fabrication [322].

Smart and adaptive hydrogel systems represent another frontier, with voxel‐programmed single‐layer hydrogels enabling rapid (≈3 s) self‐folding of 2D sheets into complex 3D configurations with multistimulus responsiveness to pH, magnetic fields, and solvents, featuring up to 500% reversible expansion and targeted drug release (up to 55%) for functional microrobots [323]. Programmable CRISPR‐Cas12a‐responsive DNA hydrogels assembled from Y‐shaped DNA motifs have enabled target‐specific payload release with responsiveness to as few as 50 genome copies, offering intelligent platforms for biosensing and therapeutic agent delivery [324].

The combination of hydrogels with smart sensors and digital health technologies may enable closed‐loop therapeutic systems capable of monitoring physiological changes and dynamically adjusting treatment responses. Emerging bioengineering strategies, including hydrogel‐mediated delivery of gene‐editing components such as CRISPR‐based systems, may provide localized and sustained genetic modulation for regenerative applications. Cell‐free therapeutic approaches based on extracellular vesicles and secretomes offer promising alternatives to conventional cell transplantation by reducing manufacturing complexity and regulatory challenges while preserving biological activity. Future platforms may also benefit from systems biology and network medicine approaches, allowing simultaneous regulation of multiple pathological pathways rather than targeting isolated mechanisms. By integrating immunomodulation, metabolic regulation, and microenvironmental control, next‐generation multifunctional hydrogels could provide more comprehensive solutions for complex tissue injuries and chronic diseases. Successful translation will depend not on increasing material complexity, but on developing clinically meaningful, manufacturable, and intelligently designed hydrogel systems that balance biological performance with translational feasibility.

### 8.8 Controversies and Unresolved Questions in Hydrogel Design

Despite remarkable advances in multifunctional hydrogel engineering, several fundamental questions remain unresolved. Many established concepts in biomaterials research require critical reassessment as the field moves from material development toward clinically meaningful applications. Addressing these controversies is essential to establish more predictive design principles and improve the translation of multifunctional hydrogel technologies.

#### 8.8.1 Beyond the Stiffness‐Centric Paradigm

Matrix stiffness has long been considered a dominant regulator of cell fate and tissue regeneration. However, emerging evidence indicates that cellular responses are also strongly influenced by dynamic mechanical properties, including stress relaxation, viscoelasticity, and network remodeling kinetics [325]. Hydrogels exhibiting similar elastic moduli but distinct relaxation behaviors can induce different cellular outcomes, suggesting that equilibrium stiffness alone cannot fully explain mechanobiological regulation. This observation challenges the traditional approach of designing hydrogels primarily by matching tissue stiffness. Importantly, increasing crosslink density to enhance stiffness simultaneously affects molecular diffusion, degradation, and viscoelastic behavior, making it difficult to isolate individual mechanical parameters [78]. Recent efforts toward independently controlling stiffness and stress relaxation have demonstrated that dynamic mechanical regulation is a critical determinant of stem cell differentiation, immune responses, and tissue remodeling [325, 326]. Therefore, future hydrogel design should move beyond static mechanical matching toward adaptive mechanical environments that better reproduce the dynamic characteristics of native ECMs.

#### 8.8.2 Reconsidering the Simplified View of Immune Regulation

Immunomodulation has become a central strategy in regenerative hydrogel development, particularly through the regulation of macrophage responses. However, the conventional M1/M2 polarization model represents an oversimplified interpretation of macrophage biology. In vivo, macrophages exist along a highly dynamic spectrum of phenotypes, with overlapping functional states influenced by tissue type, disease stage, and local environmental signals [326]. Although promoting an anti‐inflammatory M2‐like phenotype is frequently associated with improved regeneration, excessive or premature immune suppression may impair essential inflammatory processes required for debris clearance and tissue remodeling [327]. Furthermore, macrophage responses induced by hydrogel properties, including surface chemistry, degradation products, and mechanical cues, remain incompletely understood. Therefore, future immunomodulatory hydrogels should focus not on inducing a specific macrophage phenotype but on precisely regulating immune function according to the temporal and spatial requirements of tissue regeneration.

#### 8.8.3 The In Vitro–In Vivo Translation Gap

A major limitation in hydrogel research is the poor correlation between in vitro performance and in vivo therapeutic outcomes. Many studies rely on 2D cell culture models that fail to reproduce the 3D architecture, mechanical environment, immune interactions, and biochemical complexity of injured tissues [328]. Even advanced 3D culture systems often lack the dynamic conditions present during regeneration, including vascularization, inflammatory responses, and ECM remodeling [329]. Furthermore, commonly used animal models do not always accurately predict human responses due to differences in immune regulation, tissue structure, and healing mechanisms. For example, rodent wound healing relies significantly on contraction, whereas human healing depends more strongly on re‐epithelialization and granulation tissue formation [277, 330]. These limitations highlight the need for more clinically relevant models, including advanced organoid systems, large‐animal models, and standardized evaluation protocols that better predict translational outcomes.

#### 8.8.4 The Complexity–Translation Paradox

One of the major challenges in multifunctional hydrogel development is the apparent inverse relationship between material complexity and clinical translation. While increasingly sophisticated systems incorporating multiple drugs, cells, nanoparticles, and stimuli‐responsive elements demonstrate impressive preclinical performance, relatively simple hydrogels based on collagen, alginate, or HA remain among the most clinically successful platforms [331]. This discrepancy reflects the practical challenges associated with complex formulations, including manufacturing reproducibility, quality control, sterilization, regulatory classification, and cost. The addition of biological components such as growth factors or cells further increases regulatory complexity and raises concerns regarding stability, immunogenicity, and large‐scale production [310, 332]. Therefore, multifunctionality should not be pursued through unnecessary material complexity but through rational integration of functions that provide clear therapeutic advantages. The most successful future hydrogel systems may not be the most sophisticated, but rather those achieving an optimal balance between biological performance and clinical feasibility.

#### 8.8.5 Challenges in Therapeutic Factor Delivery

Although hydrogel‐based delivery of growth factors has been extensively investigated, clinical translation remains limited. The therapeutic efficacy of growth factors depends strongly on dosage, timing, spatial distribution, and interactions with the surrounding microenvironment [333]. Conventional sustained‐release approaches often fail to reproduce the highly regulated presentation of signaling molecules observed in native extracellular matrices. Additional challenges include enzymatic degradation, insufficient control over release kinetics, and the context‐dependent effects of growth factors, where identical molecules may produce different outcomes depending on tissue conditions [138, 139]. These limitations have encouraged the development of more sophisticated strategies, including affinity‐based delivery systems, ECM‐inspired binding approaches, and cell‐free therapies based on secretomes and extracellular vesicles. Such approaches may provide more complex and physiologically relevant signaling environments compared with single‐factor delivery.

#### 8.8.6 Biological Consequences of Hydrogel Degradation

The degradation behavior of hydrogels is a critical determinant of long‐term safety and regenerative performance; however, the biological effects of degradation products remain insufficiently investigated. Natural polymers generally degrade into biologically compatible molecules, although their degradation fragments may still influence inflammation and cellular signaling. Synthetic polymers may generate acidic byproducts or residual chemical components that alter the local microenvironment and potentially affect tissue responses [334]. Furthermore, incomplete removal of crosslinking agents, unreacted monomers, or photoinitiator residues represents an additional concern for long‐term implantation [335]. Comprehensive characterization of degradation products, including their chemical identity, concentration, and biological effects, is therefore essential for improving safety evaluation and regulatory acceptance.

These unresolved questions should not be considered barriers to multifunctional hydrogel development but rather opportunities to establish more rigorous and predictive design strategies. Future progress will require integrated evaluation of hydrogel properties under clinically relevant conditions, improved understanding of structure–property–function relationships, and early consideration of manufacturing and regulatory requirements. By balancing biological complexity with translational feasibility, multifunctional hydrogels can evolve from promising experimental materials into clinically impactful regenerative technologies.

## 9 Conclusion

Multifunctional hydrogels represent a transformative class of biomaterials at the interface of materials science, biology, and clinical medicine, providing new opportunities to address the complex requirements of tissue regeneration. Unlike conventional hydrogels that primarily serve as passive supports or delivery reservoirs, next‐generation multifunctional platforms integrate structural, biochemical, and therapeutic functions to actively regulate the regenerative microenvironment. The evolution of hydrogel systems from simple protective materials to advanced multifunctional platforms has been driven by progress in polymer chemistry, crosslinking technologies, fabrication strategies, and understanding of cell–material interactions. Rational design principles, including polymer selection, network architecture, mechanical tuning, degradation regulation, and biointerface engineering, determine hydrogel performance and biological outcomes. Natural polymers provide inherent bioactivity, synthetic polymers offer precise physicochemical control, and hybrid systems combine complementary advantages to overcome individual limitations. The incorporation of multiple therapeutic functions has significantly expanded the capabilities of hydrogels. Controlled delivery systems enable localized and sustained release of drugs, growth factors, nucleic acids, and nanoparticles, while cell‐laden hydrogels provide protective and instructive environments for regenerative therapies. Furthermore, immunomodulatory, antimicrobial, and stimuli‐responsive strategies allow dynamic regulation of pathological microenvironments and enhance tissue repair. These multifunctional characteristics have facilitated applications across diverse regenerative fields, including chronic wound healing, bone and cartilage repair, neural regeneration, and cardiovascular tissue engineering. Despite these remarkable advances, clinical translation remains limited by challenges associated with scalable manufacturing, sterilization, reproducibility, regulatory approval, and cost‐effectiveness. Future progress will require standardized characterization methods, predictive structure–property–function relationships, clinically relevant evaluation models, and closer collaboration among material scientists, clinicians, and industry partners. Emerging technologies, including artificial intelligence‐assisted material design, advanced manufacturing, bioprinting, and adaptive hydrogel systems, are expected to further accelerate the development of personalized regenerative therapies. By integrating fundamental biomaterial principles with translational considerations, multifunctional hydrogels hold significant promise as next‐generation platforms capable of addressing complex tissue injuries and advancing the future of regenerative medicine.

## Author Contributions

**Linyang Song**: writing – review and editing, conceptualization, visualization. **Epiphane K. Silli**: writing – review and editing, writing – original draft, conceptualization, visualization, illustrations. **Yazhen Xu**: writing – review and editing, visualization. **Huixia Lv**: supervision, writing – review and editing, project administration. **Ruoxian Song**: supervision, writing – review and editing, project administration. All authors have read and agreed to the published version of the manuscript.

## Funding

This work was financially supported by the State Key Laboratory of Drug Regulatory Science Project (2026SKLDRS3081) and the Organized Scientific Research and Development (the Cosmetic Tract) of China Pharmaceutical University (3342400034).

## Conflicts of Interest

The authors declare no conflicts of interest.

## Ethics Statement

The authors have nothing to report.

## Data Availability

No datasets were generated or analyzed during the current study.
